# Randomized Trial to Assess the Clinical Utility of Renal Allograft Monitoring by Urine CXCL10 Chemokine

**DOI:** 10.1681/ASN.0000000000000160

**Published:** 2023-05-25

**Authors:** Patricia Hirt-Minkowski, Joelle Handschin, Susanne Stampf, Helmut Hopfer, Thomas Menter, Lisa Senn, Gideon Hönger, Caroline Wehmeier, Patrizia Amico, Jürg Steiger, Michael Koller, Michael Dickenmann, Stefan Schaub

**Affiliations:** 1 Clinic for Transplantation Immunology and Nephrology, University Hospital Basel, Basel, Switzerland; 2 Molecular Immune Regulation, Department of Biomedicine, University of Basel, Basel, Switzerland; 3 Department of Pathology, University Hospital Basel, Basel, Switzerland; 4 HLA-Diagnostics and Immunogenetics, Department of Laboratory Medicine, University Hospital Basel, Basel, Switzerland

**Keywords:** rejection, transplantation

## Abstract

**Significance Statement:**

This study is the first randomized controlled trial to investigate the clinical utility of a noninvasive monitoring biomarker in renal transplantation. Although urine CXCL10 monitoring could not demonstrate a beneficial effect on 1-year outcomes, the study is a rich source for future design of trials aiming to explore the clinical utility of noninvasive biomarkers. In addition, the study supports the use of urine CXCL10 to assess the inflammatory status of the renal allograft.

**Background:**

Urine CXCL10 is a promising noninvasive biomarker for detection of renal allograft rejection. The aim of this study was to investigate the clinical utility of renal allograft monitoring by urine CXCL10 in a randomized trial.

**Methods:**

We stratified 241 patients, 120 into an intervention and 121 into a control arm. In both arms, urine CXCL10 levels were monitored at three specific time points (1, 3, and 6 months post-transplant). In the intervention arm, elevated values triggered performance of an allograft biopsy with therapeutic adaptations according to the result. In the control arm, urine CXCL10 was measured, but the results concealed. The primary outcome was a combined end point at 1-year post-transplant (death-censored graft loss, clinical rejection between month 1 and 1-year, acute rejection in 1-year surveillance biopsy, chronic active T-cell–mediated rejection in 1-year surveillance biopsy, development of *de novo* donor-specific HLA antibodies, or eGFR <25 ml/min).

**Results:**

The incidence of the primary outcome was not different between the intervention and the control arm (51% versus 49%; relative risk (RR), 1.04 [95% confidence interval, 0.81 to 1.34]; *P* = 0.80). When including 175 of 241 (73%) patients in a per-protocol analysis, the incidence of the primary outcome was also not different (55% versus 49%; RR, 1.11 [95% confidence interval, 0.84 to 1.47]; *P* = 0.54). The incidence of the individual end points was not different as well.

**Conclusions:**

This study could not demonstrate a beneficial effect of urine CXCL10 monitoring on 1-year outcomes (ClinicalTrials.gov_NCT03140514).

## Graphical abstract

**Figure:**
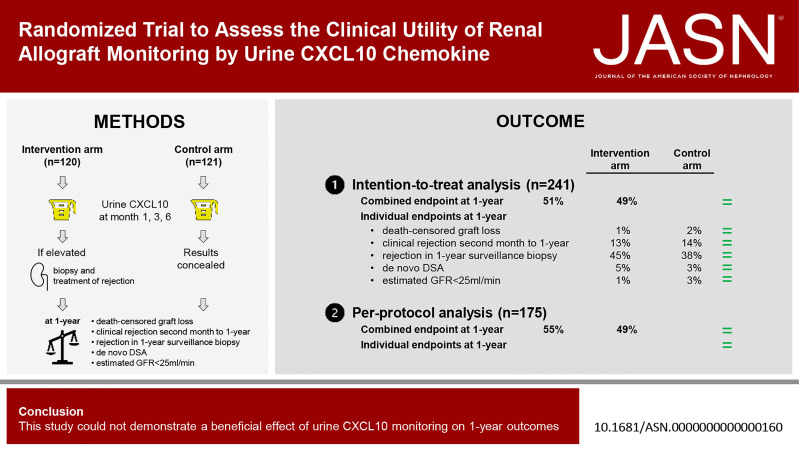

## Introduction

Renal transplantation is the preferred modality to treat end-stage kidney disease for many patients. Despite significant improvements to control the alloimmune response by immunosuppression, rejection is still the leading cause for allograft failure.^1–4^ In addition to clinical rejection episodes (*i.e.*, with concurrent rise in serum creatinine), presumably less harmful rejection processes not detectable by serum creatinine (*i.e.*, subclinical rejection) can be observed in 10%–50% of renal allograft recipients within the first year post-transplant.^5–7^ These smoldering rejection processes are still clinically relevant and might be detectable by novel noninvasive biomarkers.^5,8,9^

The CXCL10 chemokine is secreted by various leukocytes and tubular epithelial cells in the kidney, mainly induced by *γ*-interferon, a key cytokine in the inflammatory response to an allograft or infections.^10–14^ CXCL10 can be measured in the urine, and its levels have been shown to correlate with renal allograft rejection and BK polyomavirus (BKPyV) infection in several pediatric and adult renal transplant cohorts indicating its potential as a promising noninvasive biomarker.^15–23^

Biomarker development usually consists of three steps: (*1*) biomarker discovery, (*2*) biomarker validation, and (*3*) assessment of the biomarkers' clinical utility. The last step is crucial, but also very challenging, because it requires a randomized controlled trial to gather the best evidence.^24^ So far, such a study has not been performed in renal transplantation, although many noninvasive biomarkers have been proposed and validated.^25–30^ Therefore, the aim of this study was to investigate the clinical utility of renal allograft monitoring by urine CXCL10 in a prospective trial.

## Methods

### Study Protocol

**Figure 1:**
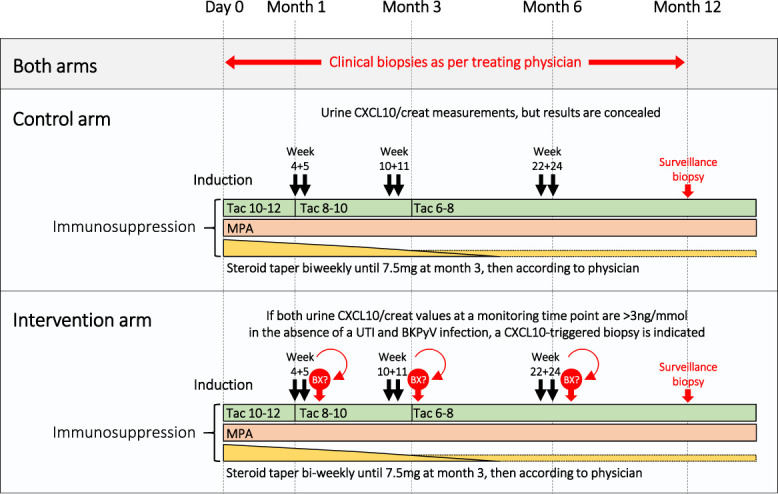
**Study design.** The target Tac trough levels are given for specific time frames post-transplant. BKPyV, polyomavirus BK; MPA, mycophenolic acid; Tac, tacrolimus; UTI, urinary tract infection. Figure 1 can be viewed in color online at www.jasn.org.

The study was approved by the local ethics committee (EKNZ 2017-00742), and all participating patients gave written informed consent. The clinical and research activities being reported are consistent with the Principles of the Declaration of Istanbul as outlined in the “Declaration of Istanbul on Organ Trafficking and Transplant Tourism.” The study protocol is detailed in Figure 1. In brief, patients were randomized into an intervention and a control arm. In both arms, two consecutive urine CXCL10 analyses were made at three specific time points (week 4 and 5, week 10 and 11, and week 22 and 24). In the intervention arm, a renal allograft biopsy was indicated, if both urine CXCL10 measurements at one specific time point were above a predefined cutoff (≥3 ng/mmol creatinine) in the absence of confounders (urinary tract infection [UTI] or BKPyV replication). Urine analyses were performed at every visit, and UTI was defined by standard criteria (urine leukocytes, urine culture, and clinical symptoms).^31^ Screening for BKPyV was performed by urine decoy cells and BKPyV DNAemia. BKPyV replication was defined by detection of ≥3 decoy cells per 10 high power fields or BKPyV DNAemia >1000 c/ml.^32^ In the control arm, urine CXCL10 analyses were also performed, but the results concealed. In both arms, clinical biopsies were performed as per treating physician, and study participants were requested for a surveillance biopsy at 12-year post-transplant. Allograft biopsies were graded by a pathologist blinded to the study arm. The study protocol included a recommendation for treatment of rejection episodes and optimization of maintenance immunosuppression, but the final decision was up to the treating physician incorporating the phenotype and severity of the rejection as well as the health status of the recipient.

The immunosuppressive regimen consisted of a maintenance therapy with tacrolimus, mycophenolic acid, and steroids. Patients with high immunological risk such as presence of donor-specific HLA antibodies (HLA-DSA) received an induction therapy with anti–T-cell globulin (anti–T-cell globulin-Fresenius or Thymoglobulin). ABO-incompatible patients received one dose of rituximab 4 weeks before the transplant, basiliximab induction, and immunoadsorption treatment depending on the antiblood group antibody titers.^33^ All other patients were considered as immunological standard risk and received basiliximab induction.^34^ In standard-risk patients having a rejection-free course, steroids were withdrawn beyond the third month post-transplant.

### Patients

All adult kidney transplant recipients able to give written informed consent and to follow the study protocol were evaluated for eligibility and enrolled within the first 4 weeks post-transplant. Key exclusion criteria were HLA-identical transplantation, primary nonfunction, and prolonged delayed graft function. We used computer-generated randomization, stratified by immunological risk (standard risk versus high risk transplants), in blocks of four with an overall 1:1 allocation. Owing to the interventional nature of this trial, randomization cannot be blinded to the treating physicians or patients.

### Urine CXCL10 Measurement

Urine CXCL10 measurements were performed as described previously in detail using the MSD V-Plex Chemokine Panel 1 (MesoScale Discovery).^35^ The cutoff for a positive result (≥3 ng/mmol creatinine) was selected on the basis of a previously determined cutoff using an ELISA platform in a large cohort.^16^

### Immunological Analyses

Two-field HLA typing of 11 loci by next-generation sequencing (NGSgoMX11-3, GenDx) was available for 236 of 241 transplantations (98%). In the remaining five cases, two-field HLA typing was inferred from intermediate resolution typing data (LinkSeq, OneLambda). We used the eplet registry version 3.0 for eplet load calculations. Single HLA-antigen beads on the Luminex platform were used for all HLA-antibody analyses. HLA-DSA were determined by virtual cross-matching.

### Investigated Outcomes

The primary outcome was a combined end point at 1-year post-transplant (death-censored graft loss, clinical rejection between day 30 and 1 year, acute rejection in 1-year surveillance biopsy, chronic active T-cell–mediated rejection (TCMR) in 1-year surveillance biopsy, development of *de novo* HLA-DSA, or eGFR <25 ml/min calculated by the CKD-Epi Collaboration 2021 formula^36^). Compared with the initial study protocol from 2017 (ClinicalTrials.gov_NCT03140514), the last two end points, although anticipated to be rare, were added. In addition, all rejection phenotypes in the 1-year surveillance biopsy were included, not only TCMR. Furthermore, chronic active TCMR was used instead of the initially defined endpoint interstitial fibrosis/tubular atrophy with inflammation. These modifications were made because these end points are generally accepted as clinically relevant. The new endpoint definitions were made before analysis of the dataset. All end points were also analyzed individually. For the modified intention-to-treat analysis, only patients having an adequate 1-year surveillance biopsy were included. For the per-protocol analysis, only patients having an adequate 1-year surveillance biopsy and a complete CXCL10 monitoring set were included. In addition, patients in the intervention arm were required to have all CXCL10-triggered biopsies being performed and all CXCL10-detected rejection episodes being treated. Secondary outcomes were development of interstitial fibrosis/tubular atrophy, proteinuria, and immunosuppression-related complications (*e.g.*, major infections, metabolic side effects).

All histological analyses throughout the study period were performed using the Banff 2015 classification.^37^ Therapeutic interventions were based on the diagnosis according to the Banff 2015 classification. The Banff 2019 classification had some major changes such as the requirement for an acute *i*-score >0 for the diagnosis of the category borderline changes.^38^ We retrospectively reclassified all allograft biopsies according to the Banff 2019 classification, and we provide these data as well.

### Statistics

The sample size calculation assumed a 40% incidence of the primary outcome. We considered a 50% reduction of the primary outcome as clinically significant. With an *α*-error of 0.05 and a power of 0.80, 81 patients were required in each arm. The recruitment was better than anticipated. Therefore, we prolonged recruitment and aimed for 100 patients in each arm with 1-year surveillance biopsies increasing the statistical power to detect smaller differences. No interim analysis was made.

Categorical data are presented as count (percentage) and were analyzed by the chi-squared test or the Fisher exact test as appropriate. Continuous data are shown as median (interquartile range) and compared by the Wilcoxon rank-sum test unless stated otherwise. For all tests, a two-tailed *P*-value < 0.05 was considered to indicate statistical significance. Time-to-event analyses were performed by the Kaplan–Meier method and compared by the log-rank test. We used JMP Pro 16 and R software package 4.1.2. for statistical analyses.

## Results

### Patient Flow and Baseline Characteristics

**Figure 2:**
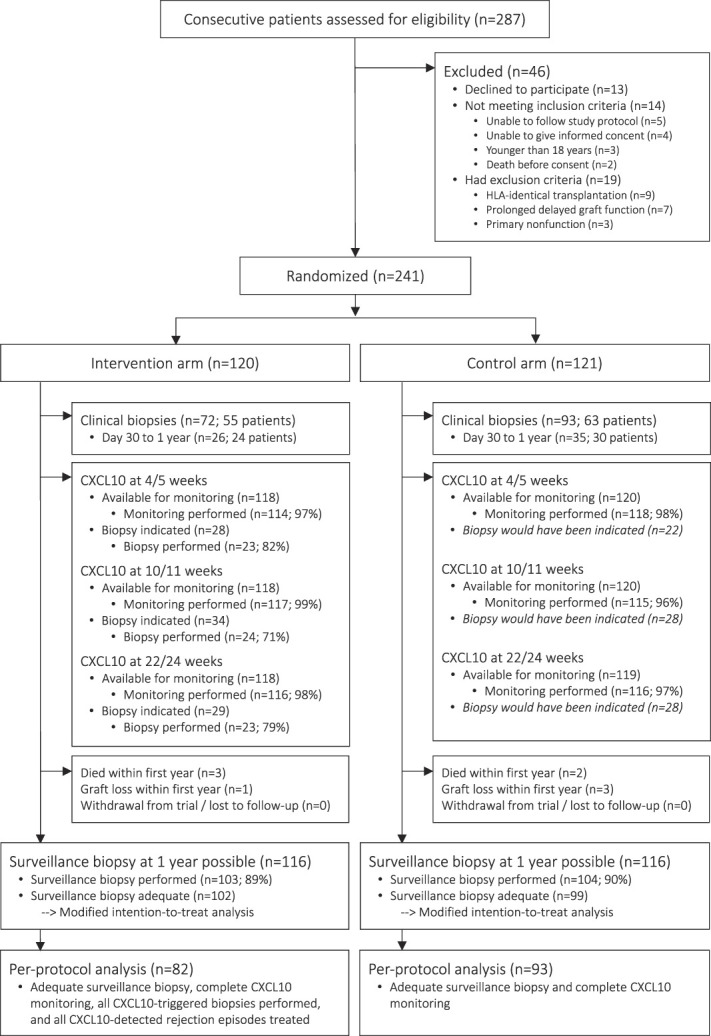
**Patient flow.** The intention-to-treat analysis included 121 patients in the intervention arm and 120 patients in the control arm. For the modified intention-to-treat analysis, only those patients having an adequate 1-year surveillance biopsy were included (intervention arm [*n*=102], control arm [*n*=99]). Finally, the per-protocol analysis included 82 patients in the intervention arm and 93 patients in the control arm.

Between September 26, 2017, and June 14, 2021, 287 patients received a kidney transplantation at the University Hospital Basel and were assessed for study eligibility (Figure 2). After exclusion of 46 patients, 241 were randomly assigned to the intervention arm (*n*=120) or the control arm (*n*=121). These 241 patients were included in the intention-to-treat analysis. Adequate 1-year surveillance biopsies were obtained in 201 patients (102 in the intervention arm, 99 in the control arm), who were evaluated in the modified intention-to-treat analysis. The per-protocol analysis included 175 patients (82 in the intervention arm, 93 in the control arm).

The patient population had a median age of 54 years, 32% were female patients, and 85% received their first renal allograft. Deceased donors accounted for 61% of all transplants, and the median donor age was 57 years. All baseline patient/donor characteristics are summarized in Table 1.

**Table 1:** Baseline characteristics

Parameter	All Patients (*n*=241)	Intervention Arm (*n*=120)	Control Arm (*n*=121)
Recipient sex female	77 (32%)	40 (33%)	37 (31%)
Recipient age	54 (44–62)	54 (44–63)	53 (44–62)
Body mass index	26.0 (22.6–29.2)	25.7 (22.3–29.5)	26.4 (22.8–29.0)
Renal disease			
ADPKD	48 (20%)	20 (17%)	28 (23%)
Diabetic nephropathy	21 (9%)	12 (10%)	9 (7%)
Glomerulonephritis	87 (36%)	45 (38%)	42 (35%)
Interstitial nephropathy	12 (5%)	7 (6%)	5 (4%)
Vascular nephropathy	25 (10%)	15 (12%)	10 (8%)
Other nephropathies	34 (14%)	15 (12%)	19 (16%)
Unknown nephropathy	14 (6%)	6 (5%)	8 (7%)
Renal replacement therapy			
Preemptive transplantation	36 (15%)	18 (15%)	18 (15%)
Dialysis vintage time, yr	2.6 (1.2–4.3)	2.8 (1.3–3.9)	2.4 (0.9–4.7)
Deceased donor	147 (61%)	75 (63%)	72 (60%)
Deceased donor details			
Cold ischemia time, h	10.0 (8.1–12.8)	10.4 (8.0–13.1)	10.0 (8.2–12.4)
DCD type	44 (30%)	23 (31%)	21 (29%)
Donor age	57 (48–65)	58 (48–65)	57 (48–64)
First transplant	206 (85%)	103 (86%)	103 (85%)
Sensitizing events			
Any	111 (46%)	56 (47%)	55 (45%)
Prior kidney transplantation	35 (15%)	17 (14%)	18 (15%)
Pregnancies	59 (24%)	28 (23%)	31 (26%)
Blood transfusions	61 (25%)	31 (26%)	30 (25%)
Current cPRA (A/B/C/DRB1/DRB345/DQB1/DPB1), %	11 (0–58)	21 (0–59)	7 (0–56)
ABO incompatible	20 (8%)	10 (8%)	10 (8%)
Pretransplant HLA-DSA	30 (12%)	16 (13%)	14 (12%)
HLA-DSA characteristics	*n*=30	*n*=16	*n*=14
Number	2 (1–2)	2 (1–3)	2 (1–2)
Class (I/II/I+II)	12/11/7	8/6/2	4/5/5
Cumulative MFI	1163 (500–2032)	1163 (507–2799)	1085 (500–1419)
HLA mismatches			
A/B/C/DRB1/DRB345/DQB1/DPB1	8 (6–10)	7 (6–10)	8 (6–10)
A/B/C	4 (3–5)	4 (3–5)	4 (3–5)
DRB1/DRB345/DQB1	3 (2–4)	3 (2–4)	3 (2–4)
Eplet load			
Total eplet load	60 (45–77)	58 (45–77)	65 (44–78)
Only antibody-verified eplets	22 (16–28)	22 (16–28)	22 (17–30)
Total eplet load HLA-DQ	11 (5–16)	12 (4–16)	11 (6–16)
Total eplet load HLA-DR	14 (6–20)	13 (6–22)	14 (6–20)
CMV risk constellation			
High risk (D+/R−)	48 (20%)	20 (17%)	28 (23%)
Intermediate risk (R+)	136 (56%)	72 (60%)	64 (53%)
Low risk (D−/R−)	57 (24%)	28 (23%)	29 (24%)
Induction therapy			
ATG	40 (17%)	20 (17%)	20 (17%)
Basiliximab	201 (83%)	100 (83%)	101 (83%)
Maintenance immunosuppression			
Tac-MMF-P	169 (70%)	87 (73%)	82 (68%)
Tac-MPS-P	70 (29%)	31 (26%)	39 (32%)
Tac-Aza-P	2 (1%)	2 (1%)	—
Delayed graft function	60 (25%)	31 (26%)	29 (24%)
Duration of hospitalization, d	10 (8–15)	10 (8–16)	11 (8–15)

Data are given as count (percentage) or median (interquartile range) as appropriate. ADPKD, autosomal polycystic kidney disease; DCD, donation after circulatory death; cPRA, calculated population-reactive antibodies; MFI, mean fluorescence intensity; HLA, human leucocyte antigen; HLA-DSA, donor-specific HLA antibodies; CMV, cytomegalovirus; ATG, anti–T-cell globulin; Tac, tacrolimus; MMF, mycophenolate mofetil; MPS, mycophenolate sodium; aza, azathioprine; P, prednisone.

### Adherence to the Protocol and Monitoring Results

**Figure 3:**
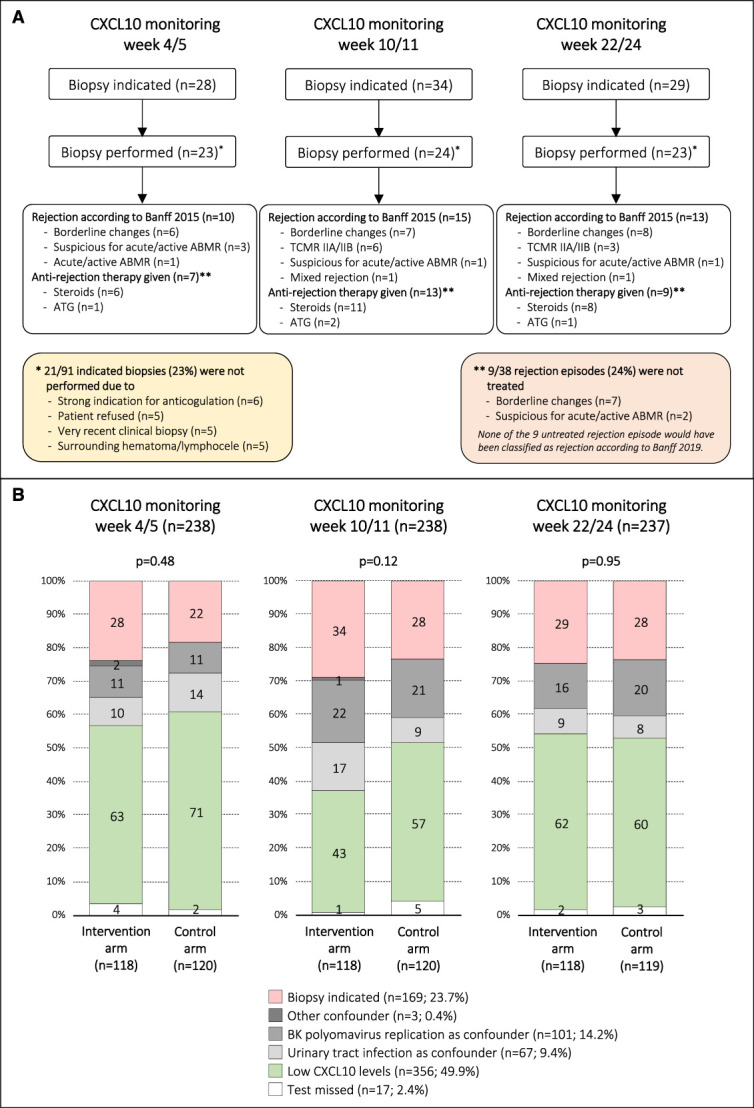
**Details of CXCL10 monitoring results and subsequent diagnostic/therapeutic interventions in the intervention arm.** (A) Summary of the diagnostic (*i.e.*, allograft biopsy) and therapeutic interventions in the intervention arm. (B) Urine CXCL10 monitoring results at the three time points, stratified by study arm. For the intervention arm, the interpretation was done prospectively; in the control arm, the interpretation was added retrospectively after study completion applying the same rules as for the intervention arm. Figure 3 can be viewed in color online at www.jasn.org.

Overall, the patients had 713 possible urine CXCL10 monitoring time points. Tests were missed in 17 of 713 occasions (2%). In the intervention group, 91 of 354 urine CXCL10 monitoring time points (26%) revealed two elevated values without confounders requiring an allograft biopsy. In 70 of 91 cases (77%), an allograft biopsy was performed. The four reasons to skip an allograft biopsy were (*1*) strong indication for anticoagulation therapy (*n*=6), (*2*) patient refusal (*n*=5), (*3*) very recent clinical biopsy (*n*=5), and (*4*) technical difficulties due to surrounding hematoma/lymphocele (*n*=5). In 38 of 70 allograft biopsies (54%), we found rejection processes; borderline changes were the most frequent phenotype (21 of 38; 55%). Antirejection therapies were given in 29 of 38 cases (76%); steroid pulses p.o. or i.v. plus optimization of maintenance immunosuppression were the most frequent therapeutic intervention (25 of 29; 86%). Full details of the rejection phenotypes and the therapeutic interventions are summarized in Figure 3A.

The results of the urine CXCL10 monitoring at the three time points were also compiled for the control arm retrospectively after completion of the study. Overall, the most frequent result was low CXCL10 levels (356 of 713; 50%), followed by biopsy indicated (169 of 713; 24%), BKPyV replication as confounder (101 of 713; 14%), and UTI as confounder (67 of 713; 9%). We observed no differences regarding the urine CXCL10 monitoring results between the two arms for all three monitoring time points (*P* = 0.48, *P* = 0.12, *P* = 0.95; for details, see Figure 3B).

### Primary and Secondary Outcomes

Using the Banff 2015 classification, we found no significant difference regarding the incidence of the primary outcome between the intervention and the control arms (51% versus 49%; RR, 1.04 [95% confidence interval, 0.81 to 1.34]; *P* = 0.80). In addition, we observed no significant differences regarding the individual end points. Furthermore, the modified intention-to-treat and the per-protocol analyses revealed no significant differences (Table 2).

**Table 2:** Primary outcomes

Using Banff 2015 Classification	Intervention Arm	Control Arm	RR (95% CI); *P* Value
Intention-to-treat analysis (*n*=241)	*n*=120	*n*=121	
Combined end point	61 (51%)	59 (49%)	1.04 (0.81 to 1.34); 0.80
Death-censored graft loss within first year	1 (1%)	3 (2%)	0.34 (0.04 to 3.19); 0.62
Clinical rejection day 30–1 yr	16 (13%)	17 (14%)	0.95 (0.50 to 1.79); 1.00
Acute rejection in 1-yr surveillance biopsy (*n*=201)	46 (45%)	38 (38%)	1.17 (0.85 to 1.63); 0.39
Chronic active TCMR in 1-yr surveillance biopsy (*n*=201)	6 (6%)	8 (8%)	0.73 (0.26 to 2.02); 0.59
*De novo* HLA-DSA at 1 yr (*n*=232)	6 (5%)	4 (3%)	1.50 (0.43 to 5.18); 0.75
eGFR <25 ml/min at 1 yr (*n*=232)	1 (1%)	4 (3%)	0.25 (0.03 to 2.20); 0.37
Modified intention-to-treat analysis (*n*=201)	*n*=102	*n*=99	
Combined end point	58 (57%)	50 (51%)	1.13 (0.87 to 1.46); 0.40
Clinical rejection day 30–1 yr	13 (13%)	12 (12%)	1.05 (0.50 to 2.19); 1.00
Acute rejection in 1-yr surveillance biopsy	46 (45%)	38 (38%)	1.17 (0.85 to 1.63); 0.39
Chronic active TCMR in 1-yr surveillance biopsy	6 (6%)	8 (8%)	0.73 (0.26 to 2.02); 0.59
*De novo* HLA-DSA at 1 yr	6 (6%)	3 (3%)	1.94 (0.50 to 7.55); 0.50
eGFR<25 ml/min at 1 yr	1 (1%)	2 (2%)	0.49 (0.04 to 5.27); 0.62
Per-protocol analysis (*n*=175)	*n*=82	*n*=93	
Combined end point	45 (55%)	46 (49%)	1.11 (0.84 to 1.47); 0.54
Clinical rejection day 30 to 1 yr	10 (12%)	10 (11%)	1.13 (0.50 to 2.59); 0.81
Acute rejection in 1-yr surveillance biopsy	37 (45%)	35 (38%)	1.20 (0.84 to 1.71); 0.36
Chronic active TCMR in 1-yr surveillance biopsy	4 (5%)	8 (9%)	0.57 (0.18 to 1.81); 0.38
*De novo* HLA-DSA at 1 yr	5 (6%)	3 (3%)	1.89 (0.47 to 7.67); 0.48
eGFR <25 ml/min at 1 yr	1 (1%)	1 (1%)	1.13 (0.07 to 17.85); 1.00

**Using Banff 2019 Classification**			
Intention-to-treat analysis (*n*=241)	*n*=120	*n*=121	
Combined end point	27 (23%)	29 (24%)	0.94 (0.59 to 1.49); 0.88
Clinical rejection day 30–1 yr	8 (7%)	9 (7%)	0.89 (0.36 to 2.25); 1.00
Acute rejection in 1-yr surveillance biopsy (*n*=201)	11 (11%)	9 (9%)	1.19 (0.51 to 2.74); 0.81
Modified intention-to-treat analysis (*n*=201)	*n*=102	*n*=99	
Combined end point	25 (25%)	21 (21%)	1.16 (0.69 to 1.92); 0.62
Clinical rejection day 30–1 yr	6 (6%)	5 (5%)	1.16 (0.37 to 3.69); 1.00
Acute rejection in 1-yr surveillance biopsy	11 (11%)	9 (9%)	1.19 (0.51 to 2.73); 0.81
Per-protocol analysis (*n*=175)	*n*=82	*n*=93	
Combined end point	19 (23%)	20 (22%)	1.08 (0.62 to 1.87); 0.86
Clinical rejection day 30–1 yr	5 (6%)	5 (5%)	1.13 (0.34 to 3.78); 1.00
Acute rejection in 1-yr surveillance biopsy	8 (10%)	9 (10%)	1.01 (0.41 to 2.49); 1.00

Data are given as count (percentage). *P*-values are calculated by two-tailed Fisher exact test. CI, confidence interval; TCMR, T-cell–mediated rejection; HLA-DSA, donor-specific HLA antibodies.

Next, we reclassified the clinical allograft biopsies and the 1-year surveillance biopsies according to the Banff 2019 definitions. This led to a reduction in the frequency of the primary outcome from around 50% to around 25%. Similarly, we found no significant differences between the two arms with respect to the intention-to-treat, the modified intention-to-treat, and the per-protocol analysis (Table 2).

**Figure 4:**
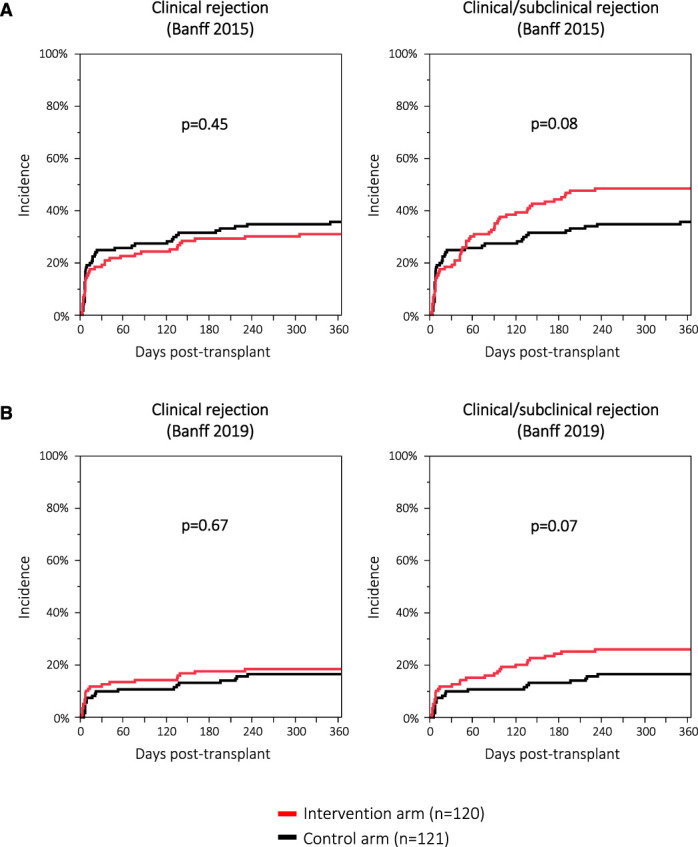
**Incidence of clinical and clinical/subclinical rejection.** (A) Defined by the Banff 2015 classification. (B) Defined by the Banff 2019 classification. Figure 4 can be viewed in color online at www.jasn.org.

Moreover, we found no significant differences regarding secondary outcomes (*e.g.*, proteinuria, BKPyV or cytomegalovirus (CMV) replication, chronic lesion scores in 1-year surveillance biopsies; Table 3). The incidence of clinical rejection (Banff 2015 classification) was not different between the intervention and the control arm (31% versus 36%; *P* = 0.45). However, the incidence of clinical or subclinical rejection defined by Banff 2015 was numerically higher in the intervention arm (48% versus 36%; *P* = 0.08) indicating that the urine CXCL10 monitoring detected additional subclinical rejection episodes (Figure 4A). The same observation was made when the Banff 2019 classification was applied (26% versus 17%; *P* = 0.07; Figure 4B). The immunosuppression exposure in the two arms was similar and within the defined range of the study protocol (Table 4).

**Table 3:** Secondary outcomes

Parameter	Intervention Arm	Control Arm	*P* Value
eGFR			
At 1 mo	47 (35–62)	50 (38–61)	0.45
At 3 mo	48 (37–64)	52 (40–61)	0.92
At 6 mo	53 (39–68)	53 (39–63)	0.88
At 1 yr	53 (43–71)	56 (44–68)	0.83
Proteinuria at 1 yr			
Protein/creatinine ratio, mg/mmol	11 (6–19)	11 (6–19)	0.82
Albumin/creatinine ratio, mg/mmol	3.0 (1.0–7.0)	2.4 (0.9–6.9)	0.65
BKPyV DNAemia	29 (24%)	26 (21%)	0.65
BKPyV peak DNAemia, copies/ml	20,200 (9000–90,450)	38,850 (6703–192,100)	0.73
Cytomegalovirus infection			
No CMV replication	90 (75%)	88 (73%)	
Asymptomatic CMV infection	20 (17%)	20 (16%)	0.80
CMV disease (CMV syndrome)	3 (2%)	6 (5%)	
CMV disease (tissue invasive)	7 (6%)	7 (6%)	
Number of other infection episodes			
Including lower UTIs (0/1/≥2)	49%/33%/18%	46%/23%/31%	0.06
Excluding lower UTIs (0/1/≥2)	58%/33%/9%	52%/27%/21%	0.04
Metabolism			
Body mass index at 1 yr	25.7 (23.1–29.4)	26.0 (23.3–30.1)	0.77
Change of body mass index from pretransplant	+0.2 (−1.0 to +1.5)	+0.6 (−0.7 to +2.1)	0.25
New-onset diabetes after transplant	9 (8%)	11 (9%)	0.82
Chronic lesions in allograft biopsies			
ci + ct score in implantation biopsy (*n*=210)	0.83±0.75	0.94±0.76	0.28
ci + ct score in 1-yr surveillance biopsy (*n*=201)	1.99±1.31	1.91±1.28	0.66
Delta ci + ct score (*n*=173)	1.21±1.34	1.17±1.44	0.85

Data are given as count (percentage), median (interquartile range), or mean±SD as appropriate. Comparisons were performed by the Fisher exact test, Wilcoxon test, or *t* test as appropriate. BKPyV, BK polyomavirus; CMV, cytomegalovirus; UTI, urinary tract infection.

**Table 4:** Immunosuppression exposure

Parameter	Intervention Arm	Control Arm	*P* Value
Tacrolimus trough levels at 1 mo, *µ*g/L	10.3 (8.8–11.5)	10.3 (8.7–12.0)	0.80
Tacrolimus trough levels at 3 mo, *µ*g/L	8.8 (7.3–10.3)	8.5 (7.3–9.8)	0.46
Tacrolimus trough levels at 6 mo, *µ*g/L	6.8 (5.7–8.1)	7.0 (6.2–7.9)	0.39
Tacrolimus trough levels at 1 yr, *µ*g/L	6.3 (5.5–7.4)	6.9 (5.7–8.0)	0.08
Mycophenolic acid trough levels at 1 mo, mg/L	2.8 (1.9–4.4)	3.1 (1.9–4.3)	0.71
Mycophenolic acid trough levels at 3 mo, mg/L	2.7 (1.7–4.3)	2.8 (1.8–4.3)	0.93
Mycophenolic acid trough levels at 6 mo, mg/L	2.1 (1.5–3.5)	2.3 (1.4–3.3)	0.84
Mycophenolic acid trough levels at 1 yr, mg/L	2.2 (1.4–3.3)	2.4 (1.5–3.7)	0.42
Triple immunosuppression at 1 yr	60 (52%)	53 (46%)	0.43

Data are given as count (percentage) or median (interquartile range) as appropriate. Comparisons were performed by the Fisher exact test or the Wilcoxon test.

In a sensitivity analysis including only 185 immunological standard risk or 56 immunological high-risk patients, no significant differences in the outcomes were observed (data not shown).

### Ancillary Analyses

**Figure 5:**
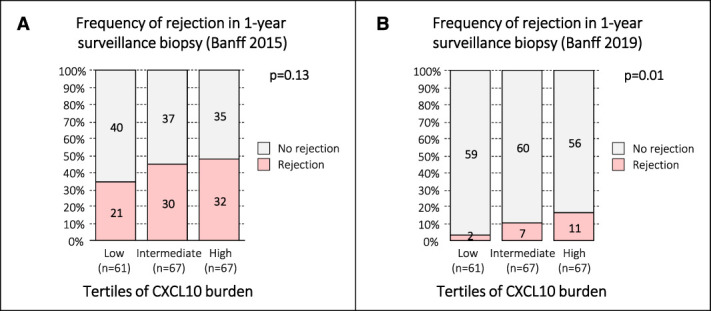
**Correlation of CXCL10 burden with occurrence of rejection in the 1-year surveillance biopsy.** Overall, patients had six urine CXCL10 monitoring checkpoints. Those having 5 or 6 measurements were included to calculate the CXCL10 burden (*n*=227; mean of 5 or 6 measurement). These 227 patients were then divided into three CXCL10 tertiles (low, intermediate, high). Of these 227 patients, 195 had 1-year surveillance biopsies (low [*n*=61], intermediate [*n*=67], high [*n*=67]). (A) Correlation of tertiles of CXCL10 burden with subsequent rejection in 1-year surveillance biopsies using the Banff 2015 classification including borderline changes (Cochran–Armitage trend test *P* = 0.13). (B) Correlation of tertiles of CXCL10 burden with subsequent rejection in 1-year surveillance biopsies using the Banff 2019 classification including borderline changes (Cochran–Armitage trend test *P* = 0.01). Figure 5 can be viewed in color online at www.jasn.org.

Because urine CXCL10 values often reflect the tissue inflammation in the allograft, we investigated the cumulative inflammatory burden assessed by urine CXCL10 with rejection in the 1-year surveillance biopsy. Indeed, tertiles of urine CXCL10 burden, calculated as the mean of measurements at the three monitoring time points, significantly correlated with rejection in 1-year surveillance biopsies defined by the Banff 2019 classification (*P* = 0.01), but not by the less stringent Banff 2015 classification (*P* = 0.13; Figure 5, A and B).

**Figure 6:**
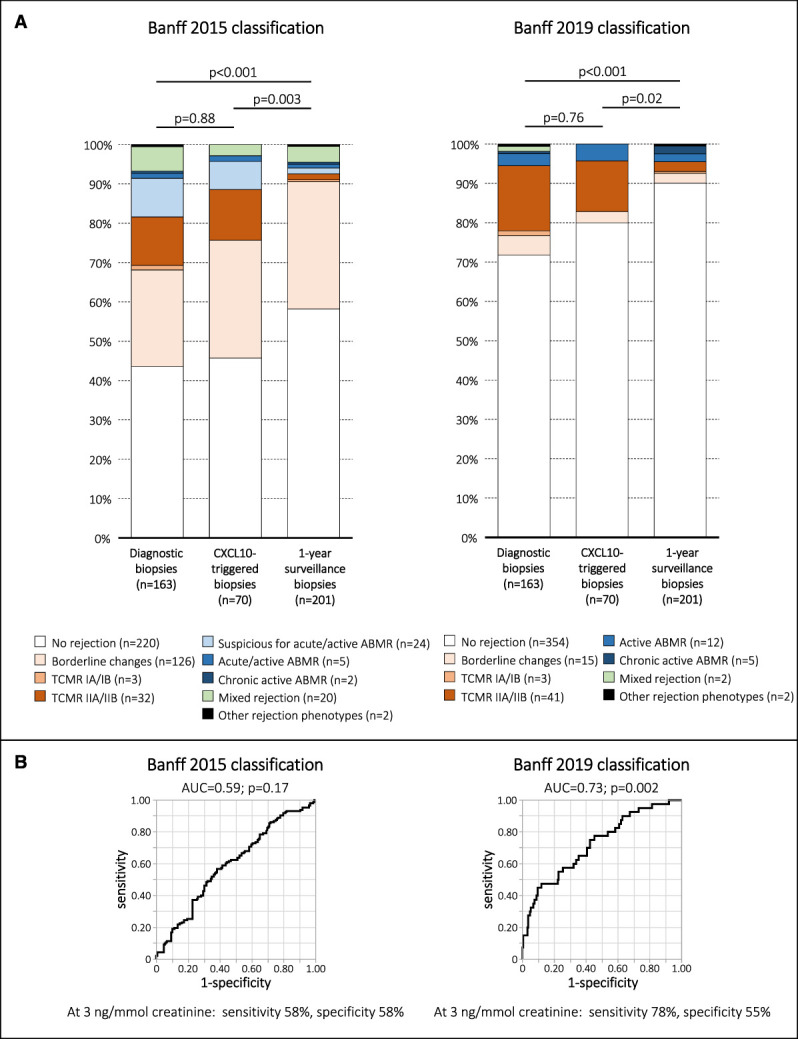
**Distribution of rejection phenotypes and diagnostic characteristics of urine CXCL10.** (A) Rejection phenotypes among diagnostic, CXCL10-triggered, and 1-year surveillance biopsies defined by the Banff 2015 and Banff 2019 classifications. (B) Diagnostic characteristics of urine CXCL10 for detection of rejection according to the Banff 2015 and Banff 2019 classifications. Figure 6 can be viewed in color online at www.jasn.org.

In total, 434 adequate allograft biopsies were obtained during the study, 163 diagnostic biopsies, 70 CXCL10-triggered biopsies, and 201 1-year surveillance biopsies. Urine CXCL10-triggered allograft biopsy yielded similar frequencies of rejection and similar proportions of rejection phenotypes as diagnostic biopsies (Banff 2015 classification: 54% versus 56%, *P* = 0.88; Banff 2019 classification: 20% versus 28%, *P* = 0.76). However, 1-year surveillance biopsies had a significantly lower yield of rejection than CXCL10-triggered biopsies (Banff 2015 classification: 42% versus 54%, *P* = 0.003; Banff 2019 classification: 10% versus 20%, *P* = 0.02; Figure 6A).

Next, we explored the diagnostic performance of CXCL10 to detect allograft rejection in the study cohort. Among 434 adequate allograft biopsies, we had to exclude 142 cases (no concurrent urine collected [*n*=108], UTI as confounder [*n*=8], BKPyV replication as confounder [*n*=26]). Notably, 100 of 108 (93%) missed urine collections were derived from biopsies obtained within the first 30 days post-transplant. Finally, 292 paired urine–biopsy samples were available for the receiver operating characteristic analysis. It revealed a significant result for rejection defined by the Banff 2019 classification (area under the curve [AUC]=0.73; *P* = 0.002), but not for rejection defined by the less stringent Banff 2015 classification (AUC=0.59; *P* = 0.17; Figure 6B).

## Discussion

The key observation in this study was that a urine CXCL10 monitoring strategy did not improve clinical outcomes at 1-year post-transplant. This was surprising because many retrospective studies demonstrated a good correlation of urine CXCL10 levels with allograft rejection.^15–23^ There are three possible explanations for this finding.

First, 1-year outcomes are not only dependent on the diagnostic performance of urine CXCL10 itself but also on the accuracy of the allograft biopsy to detect relevant rejection processes and the success rate of the antirejection therapy. While the allograft biopsy is still the gold standard for diagnosis of rejection, sampling error is a known limitation.^39^ Furthermore, borderline changes, which are the most common rejection phenotype in the current era of immunosuppression, are not well defined and subject to frequent discussions among expert groups.^9,37,38,40–42^ Additional diagnostic tools might help to better distinguish between harmful and clinically irrelevant cellular infiltrates in the allograft, which will also refine the clinical utility of novel biomarkers such as urine CXCL10.^43,44^ Indeed, the same histological features with high or low urine CXCL10 might represent different disease severities having a different prognosis.^45,46^ Although urine CXCL10 monitoring detected additional subclinical rejection episodes and we treated 76% of them (mostly steroids), this might not have been sufficient. Indeed, a recent meta-analysis found that 39% (range 22%–61%) of patients have persisting acute TCMR 2–9 months after antirejection treatment.^47^ Chronic active TCMR seems to be even less susceptible to treatment having a clinical response rate of only 20%.^48^

Second, we cannot exclude that some patients with high urine CXCL10, presumably due to confounders (*i.e.*, BKPyV replication or UTI), had in fact concomitant rejection. In our experience investigating 105 patients with BKPyV DNAemia, concomitant rejection occurred in 11%.^32^ Because 29 patients in the intervention arm experienced BKPyV DNAemia, three rejection episodes might have been missed. Although we assume a low incidence of missed rejection episodes overall, we acknowledge this diagnostic limitation of urine CXCL10 because no distinction can be made among different inflammatory diseases in the allograft or urinary tract.

Third, many retrospective studies might have overestimated the diagnostic characteristics of urine CXCL10. The AUC for diagnosis of Banff 2015 defined rejection was poorer than in previous studies; however, it was in a similar range using the Banff 2019 classification, which has a different threshold for definition of borderline changes (acute *i*-score must be >0) (Figure 6B). While all three explanations can contribute to the unexpected result of the study, we think that the first reason is the most important one.

Although the study could not demonstrate a benefit of a urine CXCL10 monitoring strategy on 1-year outcomes, we see important diagnostic and prognostic value in this biomarker. Patients with negative monitoring results throughout the first 6 months post-transplant had very often an uneventful clinical course and a rejection-free 1-year surveillance biopsy (Supplemental Figure 1B). Furthermore, persisting elevated CXCL10 values suggest ongoing inflammation in the allograft due to a rejection process or BKPyV replication (Supplemental Figure 1, C and D). The observation that the urine CXCL10 burden within the first 6 months post-transplant correlates with Banff 2019–defined rejection in 1-year surveillance biopsies indicates potential for prediction of more severe rejection phenotypes. In addition, CXCL10-triggered biopsies had a higher yield of rejection processes than surveillance biopsies (*i.e.*, similar to diagnostic biopsies), suggesting that urine CXCL10 monitoring is a reasonable alternative to universal performance of surveillance biopsies. Finally, instead of a single cutoff (*i.e.*, 3 ng/mmol in this study), a low and a high cutoff could be defined to rule-in or rule-out rejection more precisely.

This is the first randomized study to explore the clinical utility of a noninvasive rejection monitoring biomarker in renal transplantation. It clearly highlights major challenges of such studies and can be very helpful for the design of future trials. The key parameter driving the results of this study was the incidence of rejection, which depends on the used histological classification. In a *post hoc* analysis, the study had power of 0.80 to detect a 36% reduction of the primary outcome (Banff 2015), for the per-protocol analysis it had power of 0.80 to detect a 42% reduction of the primary outcome (Banff 2015). Therefore, the assumption for the power calculation in this trial was met, even for the per-protocol analysis. When using the Banff 2019 classification, the incidence of the primary outcome dropped to 24%. In this scenario, 260 patients in each arm would be required to detect a 40% reduction of the primary outcome (power 0.80, *α*-error 0.05). Another randomized controlled trial investigating the clinical utility of urine CXCL10 is still ongoing (ClinicalTrials.gov: NCT03206801).^49^ The investigators use an enrichment strategy to randomize 250 patients with elevated urine CXCL10 1:1 into an intervention and a control arm. This multicenter study anticipates an event rate of 55% in the control arm and can detect a reduction of 35.6% in the intervention arm (power 0.80, *α*-error 0.05).

We acknowledge several important limitations of this study. First, this study was not powered to detect clinically relevant differences when the more stringent Banff 2019 classification is applied, and it might also have missed smaller differences using the Banff 2015 criteria. Second, the assumed effect size of 50% reduction in the primary outcome was very ambitious and according to very recent reports likely difficult to reach.^47,48^ Third, the incidence of the primary outcome was largely driven by the rejection category borderline changes, whose definition was altered from the Banff 2015 to the Banff 2019 classification. The clinical relevance of borderline changes is still debated and thus might be a problematic trial end point.^50^

Advantages of this study are the broad inclusion criteria reflecting a real-life population and the high compliance with the study protocol including 1-year surveillance biopsies. However, there are also some limitations such as the single-center design and the reduced biomarker assessment density (*i.e.*, months 1, 3, and 6). This biomarker assessment schedule was selected for two reasons. First, most rejection episodes occur within the first 6 months post-transplant. Second, in this time frame, major adaptations of the immunosuppression regimen were made (steroid withdrawal and lowering of tacrolimus target trough levels). Indeed, according to the study protocol, no further reduction of immunosuppression was stipulated beyond month 6 post-transplant.

In conclusion, this study could not demonstrate a beneficial effect of a urine CXCL10 monitoring strategy on 1-year outcomes. However, urine CXCL10 can provide important information on the inflammatory status of the renal allograft. Further studies including more patients are warranted to delineate the clinical utility of urine CXCL10 monitoring in renal transplantation.

## Supplementary Material

## Disclosures

All authors have nothing to disclose.

## Funding

This study was supported by the Swiss National Science Foundation (Grant no 32003B_169310/1).

## Acknowledgments

The authors would like to thank the teams of the renal transplant outpatient clinic and the HLA laboratory, especially Simone Rychen-Hirschler, Claudia Petit, and Aynur Gubelmann.

## Author Contributions

**Conceptualization:** Michael Dickenmann, Patricia Hirt-Minkowski, Michael Koller, Stefan Schaub.

**Data curation:** Patrizia Amico, Joelle Handschin, Patricia Hirt-Minkowski, Michael Koller, Stefan Schaub, Lisa Senn, Caroline Wehmeier.

**Formal analysis:** Patrizia Amico, Joelle Handschin, Patricia Hirt-Minkowski, Helmut Hopfer, Gideon Hönger, Michael Koller, Thomas Menter, Stefan Schaub, Lisa Senn, Susanne Stampf, Caroline Wehmeier.

**Funding acquisition:** Stefan Schaub.

**Investigation:** Helmut Hopfer, Michael Koller, Thomas Menter, Stefan Schaub, Jürg Steiger, Caroline Wehmeier.

**Methodology:** Joelle Handschin, Helmut Hopfer, Gideon Hönger, Michael Koller, Thomas Menter, Stefan Schaub, Susanne Stampf.

**Project administration:** Michael Dickenmann, Joelle Handschin, Patricia Hirt-Minkowski, Stefan Schaub.

**Resources:** Patrizia Amico, Michael Dickenmann, Joelle Handschin, Patricia Hirt-Minkowski, Helmut Hopfer, Thomas Menter, Lisa Senn, Jürg Steiger, Caroline Wehmeier.

**Supervision:** Michael Dickenmann.

**Validation:** Patricia Hirt-Minkowski, Stefan Schaub.

**Visualization:** Stefan Schaub.

**Writing – original draft:** Patricia Hirt-Minkowski, Stefan Schaub.

**Writing – review & editing:** Patrizia Amico, Michael Dickenmann, Joelle Handschin, Patricia Hirt-Minkowski, Helmut Hopfer, Gideon Hönger, Michael Koller, Thomas Menter, Stefan Schaub, Lisa Senn, Susanne Stampf, Jürg Steiger, Caroline Wehmeier.

## Data Sharing Statement

The data that support the findings of this study are available from the corresponding author on reasonable request.

## Supplemental Material

This article contains the following supplemental material online at http://links.lww.com/JSN/E441 and http://links.lww.com/JSN/E442.

Supplemental Figure 1. Illustration of individual patients.

Supplemental Appendix. Original study protocol, final protocol, summary of changes. Original statistical analysis plan (protocol description), final statistical analysis plan, summary of changes.
