# Supplementary material for: Randomized Trial to Assess the Clinical Utility of Renal Allograft Monitoring by Urine CXCL10 Chemokine
Source: J Am Soc Nephrol. 2023 May 25;34(8):1456–69. doi: 10.1681/ASN.0000000000000160 (PMC10400101; doi:10.1681/ASN.0000000000000160)
Supplement: Supplementary file 1 [file jasn-34-1456-s001.pdf]

CJASN
Clinical Journal of the American Society of Nephrology

JASN
JOURNAL OF THE AMERICAN SOCIETY OF NEPHROLOGY

Kidney360

Manuscript ID:

Adapted* CONSORT Checklist for Clinical Trials

Section/Topic	Checklist Item	Response
**Title**	Identified as a randomized trial in the title.	Yes
**Background**	Specific objectives or hypotheses clearly stated.	Yes
**Trial Design**	Trial design *(such as parallel, factorial)* including allocation ratio.	Yes
	Important changes to methods after trial commencement *(such as eligibility criteria)*, with reasons.	Yes
**Participants**	Eligibility criteria for participants.	Yes
	Settings and locations where the data were collected.	Yes
**Interventions**	The interventions for each group with sufficient details to allow replication, including how and when they were actually administered.	Yes
**Outcomes**	Completely defined per-specified primary and secondary outcome measures, including how and when they were assessed.	Yes
	Any changes to trial outcomes after the trial commenced, with reasons.	Yes
**Sample Size**	How sample size was determined.	Yes
	Explanation of any interim analyses and stopping guidelines.	NA
**Randomization**		
• ***Sequence generation***	Method used to generate the random allocation sequence.	Yes
	Type of randomization; details of any restriction *(such as blocking and block size)*.	Yes
• ***Allocation concealment mechanism***	Mechanism used to implement the random allocation sequence *(such as sequentially numbered containers)*, describing any steps taken to conceal the sequence until interventions were assigned.	Yes
• ***Implementation***	Who generated the random allocation sequence, who enrolled participants, and who assigned participants to interventions.	Yes
**Blinding**	If done, who was blinded after assignment to interventions *(for example, participants, care providers, those assessing outcomes)*.	NA
	If relevant, description of the similarity of interventions.	Yes
**Statistical Methods**	Statistical methods used to compare groups for primary and secondary outcomes.	Yes
	Methods for additional analyses, such as subgroup analyses and adjusted analyses.	Yes
**Participant Flow** *(a diagram is strongly recommended)*	For each group, the numbers of participants who were randomly assigned, received intended treatment, and were analyzed for the primary outcome .	Yes
	For each group, losses and exclusions after randomization, together with reasons.	Yes
**Recruitment**	Dates defining the periods of recruitment and follow-up.	Yes
	Why the trial ended or was stopped.	Yes
**Baseline Data**	A table showing baseline demographic and clinical characteristics for each group.	Yes
**Numbers Analyzed**	For each group, number of participants *(denominator)* included in each analysis and whether the analysis was by original assigned groups.	Yes
**Outcomes and Estimation**	For each primary and secondary outcome, results for each group, and the estimated effect size and its precision *(such as 95% confidence interval)*.	Yes
	For binary outcomes, presentation of both absolute and relative effect sizes.	Yes
**Ancillary Analyses**	Results of any other analyses performed, including subgroup analyses and adjusted analyses, distinguishing pre-specified from exploratory.	Yes
**Harms**	All important harms or unintended effects in each group.	Yes
**Data Sharing**	A data sharing statement is included.	Yes

Additional Details:

*This checklist has been adapted by the ASN journals for author and editor use.

November 2021
