# Supplementary material for: Randomized Trial to Assess the Clinical Utility of Renal Allograft Monitoring by Urine CXCL10 Chemokine
Source: J Am Soc Nephrol. 2023 May 25;34(8):1456–69. doi: 10.1681/ASN.0000000000000160 (PMC10400101; doi:10.1681/ASN.0000000000000160)
Supplement: Supplementary file 2 [file jasn-34-1456-s002.pdf]

Supplemental Material

Supplemental Figure 1. Illustration of individual patients

Supplemental Appendix. Original study protocol, final protocol, summary of changes. Original Statistical Analysis plan (protocol description), final statistical analysis plan, summary of changes.

Supplementary Figure Legend

Supplementary Figure 1. Illustration of individual patients. Negative urine CXCL10 values (<3ng/mmol creatinine) are marked with a green background, positive urine CXCL10 values (≥3ng/mmol creatinine) are marked with a red background. **A:** This patient had a subclinical Banff IIB rejection, which was detected by CXCL10-monitoring. The rejection was successfully treated, and a subsequent clinical biopsy showed an isolated t1 lesion (urine CXCL10 was negative). The 1-year surveillance biopsy was without rejection and urine CXCL10 again negative. **B:** This patient had low urine CXCL10 values at all monitoring checkpoints and a 1-year surveillance biopsy without rejection. **C:** This patient had an ongoing ABMR, which was treated several times. An intermittent BKPyV viremia was observed as well. Despite anti-rejection treatment urine CXCL10 values were very high throughout the entire course, indicating ongoing rejection. The highest urine CXCL10 values were observed during concomitant BKPyV infection. **D:** This patient had high and prolonged BKPyV viremia. Interestingly, urine CXCL10 were highest when BKPyV viremia started to decline, suggesting a strong inflammatory response against the virus at this time point.

Supplementary Figure 1

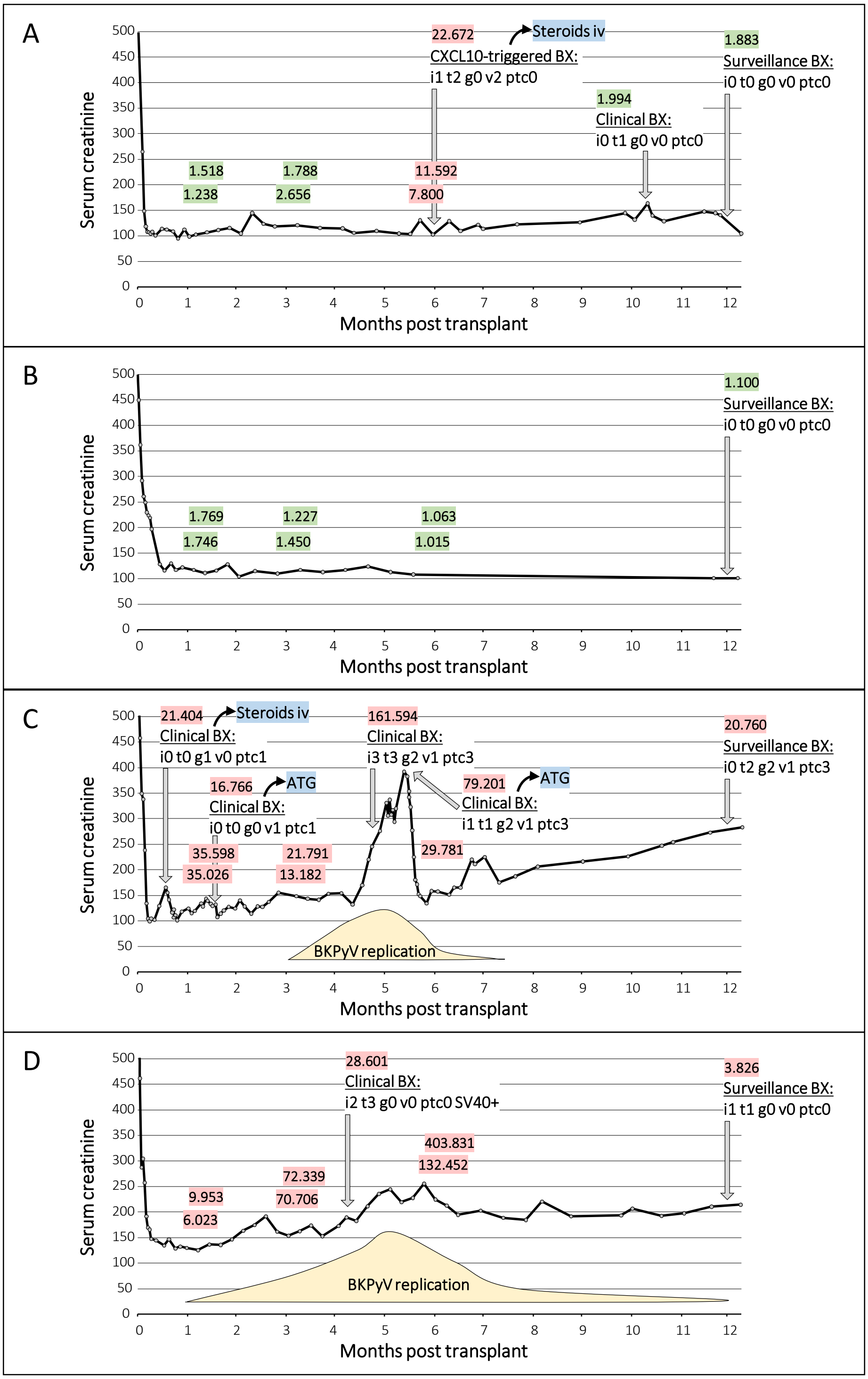

Supplemental Appendix

Randomized trial to assess the clinical utility of renal allograft monitoring by urine CXCL10 chemokine

This supplement contains the following items:

1. Original protocol, final protocol, summary of changes.

2. Original statistical analysis plan (protocol description), final statistical analysis plan, summary of changes

The study protocol including the statistical analysis plan dating from June 14, 2017 was approved by ethics committee (EKNZ; project ID 2017-00742) on June 19, 2017. This document is considered the original protocol and the original statistical analysis plan.

Before completion of the study and closure of the database, some modifications in the endpoints at one year were made (final protocol). In addition, the final statistical analysis plan was created.

Original protocol	page 2
Final protocol	page 45-46
Summary of changes	page 45-46
Original statistical analysis plan	pages 31-33
Final statistical analysis plan	page 47
Summary of changes	page 48

Clinical Study Protocol

Urine CXCL10 chemokine monitoring post-renal transplant: a single-center randomized controlled trial

Short title: Urine chemokine monitoring

Study Type:	Randomized trial investigating the effect of a urine chemokine-based intervention on clinical outcomes
Study Categorisation:	B
Study Registration:	ClinicalTrials.gov (NCT03140514)
Study Identifier:	Urine chemokine monitoring
Sponsor, Sponsor-Investigator or Principal Investigator:	University Hospital Basel,<br>Represented by the PI<br>Prof. Stefan Schaub<br>Transplantation Immunology and Nephrology<br>University Hospital Basel<br>Petersgraben 4<br>4031 Basel<br>Email: stefan.schaub@usb.ch<br>Phone: 061 265 45 33<br>Funded by SNF grant 32003B_169310 / 1
Investigational Product:	none
Protocol Version and Date:	Version 2.0 of June 14th, 2017

CONFIDENTIAL

The information contained in this document is confidential and the property of the PI. The information may not - in full or in part - be transmitted, reproduced, published, or disclosed to others than the applicable Competent Ethics Committee(s) and Regulatory Authority(ies) without prior written authorisation from the PI except to the extent necessary to obtain informed consent from those who will participate in the study.

Signature Page(s)

Study number	Study registry and registration number
Study Title	Urine CXCL10 chemokine monitoring post-renal transplant: a single-center randomized controlled trial

The Sponsor-Investigator and trial statistician have approved the protocol version 1.0 of April 25th, 2017, and confirm hereby to conduct the study according to the protocol, current version of the World Medical Association Declaration of Helsinki, ICH-GCP guidelines or ISO 14155 norm if applicable and the local legally applicable requirements.

Sponsor-Investigator: University Hospital Basel,
Represented by the PI Prof. Stefan Schaub

Basel, 14.6.17

Place/Date Signature

Trial statistician: PD Dr. Michael Koller

Basel 15.6.17

Place/Date Signature

Local Principal Investigator at study site*:

I have read and understood this trial protocol and agree to conduct the trial as set out in this study protocol, the current version of the World Medical Association Declaration of Helsinki, ICH-GCP guidelines or ISO 14155 norm and the local legally applicable requirements.

Site	Transplantation Immunology and Nephrology<br>University Hospital Basel<br>Basel, Switzerland
Principal investigator	Prof. Stefan Schaub

Basel, 14.6.17

Place/Date   Signature

Site	Transplantation Immunology and Nephrology<br>University Hospital Basel<br>Basel, Switzerland
Principal investigator	PD Dr. Helmut Hopfer

Basel, 15.6.17

Place/Date   Signature

Site	Transplantation Immunology and Nephrology<br>University Hospital Basel<br>Basel, Switzerland
Principal investigator	PD Dr. Patricia Hirt-Minkowski

Basel, 14.6.17

Place/Date   Signature

Site	Transplantation Immunology and Nephrology<br>University Hospital Basel<br>Basel, Switzerland
Principal investigator	PD Dr. Axel Regeniter

Basel 15.6.17

Place/Date   Signature

Table of Contents

STUDY SYNOPSIS **7**
ABBREVIATIONS **11**
STUDY SCHEDULE **12**
1. STUDY ADMINISTRATIVE STRUCTURE **13**
1.1 Sponsor, Sponsor-Investigator 13
1.2 Principal Investigator(s) 13
1.3 Statistician ("Biostatistician") 13
1.4 Laboratory 14
1.5 Monitoring institution 14
1.6 Data Safety Monitoring Committee 14
1.7 Any other relevant Committee, Person, Organisation, Institution 14
2. ETHICAL AND REGULATORY ASPECTS **15**
2.1 Study registration 15
2.2 Categorisation of study 15
2.3 Competent Ethics Committee (CEC) 15
2.4 Competent Authorities (CA) 15
2.5 Ethical Conduct of the Study 15
2.6 Declaration of interest 15
2.7 Patient Information and Informed Consent 15
2.8 Participant privacy and confidentiality 16
2.9 Early termination of the study 16
2.10 Protocol amendments 16
3. BACKGROUND AND RATIONALE **17**
3.1 Background and Rationale 17
3.2 Investigational Product (treatment, device) and Indication 19
3.3 Preclinical Evidence 19
3.4 Clinical Evidence to Date 19
3.5 Dose Rationale / Medical Device 20
3.6 Explanation for choice of comparator (or placebo) 20
3.7 Risks / Benefits 20
3.8 Justification of choice of study population 20
4. STUDY OBJECTIVES **20**
4.1 Overall Objective 20
4.2 Primary Objective 20
4.3 Secondary Objectives 20
4.4 Safety Objectives 20
5. STUDY OUTCOMES **20**
5.1 Primary Outcome 20
5.2 Secondary Outcomes 21
5.3 Other Outcomes of Interest 21
5.4 Safety Outcomes 21
6. STUDY DESIGN **22**
6.1 General study design and justification of design 22
6.2 Methods of minimising bias 22
6.2.1 Randomisation 22

6.2.2 Blinding procedures 22
6.2.3 Other methods of minimising bias..23
6.3 Unblinding Procedures (Code break)...23
7. STUDY POPULATION 23
7.1 Eligibility criteria 23
7.2 Recruitment and screening 23
7.3 Assignment to study groups..23
7.4 Criteria for withdrawal / discontinuation of participants...23
8. STUDY INTERVENTION 23
8.1 Identity of Investigational Products (treatment / medical device)......................................23
8.1.1 Experimental Intervention (treatment / medical device)..23
8.1.2 Control Intervention (standard/routine/comparator treatment / medical device) 24
8.1.3 Packaging, Labelling and Supply (re-supply) 24
8.1.4 Storage Conditions..24
8.2 Administration of experimental and control interventions 24
8.2.1 Experimental Intervention 24
8.2.2 Control Intervention...24
8.3 Dose / Device modifications..24
8.4 Compliance with study intervention 24
8.5 Data Collection and Follow-up for withdrawn participants 24
8.6 Trial specific preventive measures..24
8.7 Concomitant Interventions (treatments)..24
8.8 Study Drug / Medical Device Accountability 24
8.9 Return or Destruction of Study Drug / Medical Device 24
9. STUDY ASSESSMENTS.. 25
9.1 Study flow chart(s) / table of study procedures and assessments 25
9.2 Assessments of outcomes 26
9.2.1 Assessment of primary outcome...26
9.2.2 Assessment of secondary outcomes 26
9.2.3 Assessment of other outcomes of interest...26
9.2.4 Assessment of safety outcomes 26
9.2.5 Assessments in participants who prematurely stop the study 27
9.3 Procedures at each visit...27
9.3.1 Study inclusion visit...27
9.3.2 Regular visits until the end of the first year post-transplant**Fehler! Textmarke nicht definiert.**
9.3.3 One-year study visit including read-out allograft biopsy 28
9.3.4 Long-term follow-up 28
10. SAFETY 28
10.1 Drug studies 28
10.1.1 Definition and assessment of (serious) adverse events and other safety related events ...28
10.1.2 Reporting of serious adverse events (SAE) and other safety related events.....................29
10.1.3 Follow up of (Serious) Adverse Events..30
11. STATISTICAL METHODS.. 30
11.1 Hypothesis...30
11.2 Determination of Sample Size..30
11.3 Statistical criteria of termination of trial 31

11.4 Planned Analyses 31
11.4.1 Datasets to be analysed, analysis populations 31
11.4.2 Primary Analysis 31
11.4.3 Secondary Analyses 31
11.4.4 Interim analyses 32
11.4.5 Safety analysis 32
11.4.6 Deviation(s) from the original statistical plan 32
11.5 Handling of missing data and drop-outs 32
12. QUALITY ASSURANCE AND CONTROL 32
12.1 Data handling and record keeping / archiving 32
12.1.1 Case Report Forms 32
12.1.2 Specification of source documents 32
12.1.3 Record keeping / archiving 32
12.2 Data management 33
12.2.1 Data Management System 33
12.2.2 Data security, access and back-up 33
12.2.3 Analysis and archiving 33
12.2.4 Electronic and central data validation 33
12.3 Monitoring 33
12.4 Audits and Inspections 33
12.5 Confidentiality, Data Protection 33
12.6 Storage of biological material and related health data 33
13. PUBLICATION AND DISSEMINATION POLICY 33
14. FUNDING AND SUPPORT 34
14.1 Funding 34
14.2 Other Support 34
15. INSURANCE 34
16. REFERENCES 35
17. APPENDICES 38

STUDY SYNOPSIS

**Sponsor / Sponsor-Investigator**	University Hospital of Basel<br>Prof. Stefan Schaub, Principal Investigator<br>Funded by SNF grant 32003B_169310 / 1
**Study Title:**	Urine CXCL10 chemokine monitoring post-renal transplant: a single-center randomized controlled trial
**Short Title / Study ID:**	Urine Chemokine monitoring
**Protocol Version and Date:**	Version 2.0 of June 1st, 2017
**Trial registration:**	ClinicalTrials.gov (NCT03140514)
**Study category and Rationale**	B<br>In this study we investigate whether early treatment of rejection, as detected by urine CXCL10, improves renal allograft outcomes.
**Clinical Phase:**	Other clinical trial
**Background and Rationale:**	Renal replacement therapy due to end-stage renal disease is common (~3700 Swiss are currently on dialysis) and costly (~100'000 CHF/patient/year). Transplantation is the therapy of choice for many patients due to improved survival, better quality of life and it has significant cost-savings after the first year post-transplant compared to dialysis (~80'000 CHF/patient/year).<br>A major challenge in transplantation is how to optimize anti-rejection therapy to balance the risk of rejection from under-immunosuppression against the risk of infections/cancer from over-immunosuppression. The ideal regimen would provide the minimum therapy to avoid complications while being sufficient to prevent rejection, which accounts for ~50% of death-censored allograft failures. In the 1st year post-transplant, 30% patients have rejection of which 2/3 is not detected by currently used standard-of-care tests (i.e. serum creatinine, proteinuria). Accurate non-invasive tests are required so that rejection can be treated early and anti-rejection therapy optimized.<br>We have identified new non-invasive urine tests to detect early pre-clinical rejection (i.e. CXCL10 chemokine) and to predict long-term outcomes in kidney transplantation (i.e. CXCL10 and CCL2 chemokine). We showed in several retrospective studies that urine CXCL10 detects rejection better than standard-of-care tests. Clearly, the next step is to investigate whether an early treatment strategy based on urine CXCL10-monitoring improves outcomes in a prospective study.
**Objective(s):**	1) To determine the effectiveness of early treatment of rejection, as detected by urine CXCL10, to improve graft outcomes.<br>2) To investigate the urine CXCL10 kinetics in response to anti-rejection therapy<br>3) To evaluate and independently validate different novel diagnostic and prognostic markers for rejection or long-term outcomes (exploratory objective).

**Outcome(s):**	Ad 1)<br>**The primary 1-year composite outcome will consist of at least one of the following four outcomes:**<br>- Graft loss not due to death of the patient, or<br>- Biopsy-proven clinical acute rejection from 4-weeks to 1-year post-transplant, or<br>- Subclinical T-cell mediated rejection in 1-year surveillance biopsy defined by t>0 and/or v>0, or<br>- Interstitial fibrosis / tubular atrophy with inflammation (IFTA+i defined by the Mayo Clinic criteria) in 1-year surveillance biopsy<br><br>**Secondary outcomes:**<br>**- Efficacy**:<br>- Microvascular inflammation at 1-year (ptc, g, c4d, cg)<br>- Development of IFTA from implantation to 1-year (Δ ci, ct, cv)<br>- Days from transplantation to biopsy-proven clinical acute rejection<br>- Proteinuria >500mg/day at 6- and 12-months post-transplant<br>**- Safety**:<br>- Number of total, indication and CXCL10-triggered biopsies within the first year post-transplant<br>- Biopsy-related complications within the first year post-transplant<br>- Immunosuppression-related complications (infections, cancer) within the first year post-transplant<br>**- Long-term outcomes:**<br>- Graft loss including its cause<br>- Death including its cause<br>- Allograft function (creatinine and eGFR)<br>- Proteinuria<br>- Biopsy-proven rejection<br><br>Ad 2)<br>Correlation of histological evolution of rejection with changes of CXCL10 chemokine levels.<br><br>Ad 3)<br>Prospectively validate urine CXCL10 & CCL2 as well as novel biomarkers as predictors for rejection and long-term outcomes.
**Study design:**	Single-center parallel-group randomized controlled trial.

**Inclusion / Exclusion criteria:**	Inclusion criteria:<br>- All consenting adult (age >=18 years) renal allograft recipients<br><br>Exclusion criteria:<br>- HLA-identical living donor transplantation<br>- Primary non-function<br>- Participation in immunosuppression interventional trials<br><br>Patients will be stratified according to their immunological risk into two groups, which will be randomized and analysed separately.<br>- Normal risk transplants (i.e. no immunological risk as detailed below)<br>- High risk transplants defined as<br>• ABO-incompatible and/or<br>• presence of donor-specific HLA-antibodies and/or<br>• husband-to-wife transplant with shared children or child-to-mother transplant
**Measurements and procedures:**	The study is designed as a two-arm parallel-group RCT. In both arms urine chemokines will be measured at specific time points. Physician-initiated allograft biopsies can be performed in both arms at any time. In both arms, a readout allograft biopsy will be performed at one year post-transplant.
**Study Product / Intervention:**	In the intervention arm, sustained elevated urine CXCL10 levels will trigger the performance of an additional allograft biopsy (=CXCL10-triggered biopsy). If a rejection process is detected, it will be treated.
**Control Intervention (if applicable):**	In the control arm, urine CXCL10 levels will be measured and concealed.
**Number of Participants with Rationale:**	For normal risk patients: n=178<br>- interventional arm: n=89<br>- control arm: n=89<br>For high risk patients: n=60<br>- interventional arm: n=30<br>- control arm: n=30
**Study Duration:**	5 years
**Study Schedule:**	First patient in: 09/2017<br>Last patient out: 09/2022

**Investigator(s):**	Prof. Stefan Schaub<br>Transplantation Immunology and Nephrology<br>University Hospital Basel<br>Petersgraben 4<br>4031 Basel<br>Email: stefan.schaub@usb.ch<br>Phone: 061 265 45 33<br><br>PD Dr. Patricia Hirt-Minkowski<br>Transplantation Immunology and Nephrology<br>University Hospital Basel<br>Petersgraben 4<br>4031 Basel<br>Email: patricia.hirt-minkowski@usb.ch<br>Phone: 061 556 56 22<br><br>PD Dr. Helmut Hopfer<br>Institute of Pathology<br>University Hospital Basel<br>Petersgraben 4<br>4031 Basel<br>Email: helmut.hopfer@usb.ch<br>Phone: 061 328 78 90<br><br>PD Dr. Axel Regeniter<br>Department of Laboratory Medicine<br>University Hospital Basel<br>Petersgraben 4<br>4031 Basel<br>Email: axel.regeniter@usb.ch<br>Phone: 061 328 62 29
**Study Centre(s):**	Single-centre: University Hospital Basel
**Statistical Considerations:**	For normal risk transplants:<br>We conservatively estimate a 40% incidence of the primary outcome. We consider a 50% reduction of the primary outcome as clinically significant. Sample size estimates were based on two-sample test of proportions using JMP 12 statistical software. With an alpha error of 0.05 and a power of 0.80, 81 patients are required in each arm. Assuming a 10% drop-out rate, we need to enroll 178 patients. This corresponds to a recruitment phase of approximately 4 years in our center, given an average normal risk transplantation rate of 45 per year.<br>For high risk transplants:<br>Within the 4 year recruitment phase we will include approximately 60 high risk transplants. We will gain important data in this population for future trial design. However, we might lack statistical power for the same evaluation as for normal risk transplants. Therefore, we consider this subgroup as a pilot study.
**GCP Statement:**	This study will be conducted in compliance with the protocol, the current version of the Declaration of Helsinki, the ICH-GCP or ISO EN 14155 (as far as applicable) as well as all national legal and regulatory requirements.

ABBREVIATIONS

Provide a list of abbreviations used on the protocol - to be completed

AE	Adverse Event
CA	Competent Authority (e.g. Swissmedic)
CEC	Competent Ethics Committee
CRF	Case Report Form
ClinO	Ordinance on Clinical Trials in Human Research *(in German: KlinV, in French: OClin)*
eCRF	Electronic Case Report Form
CTCAE	Common terminology criteria for adverse events
DSUR	Development safety update report
GCP	Good Clinical Practice
IB	Investigator's Brochure
Ho	Null hypothesis
H1	Alternative hypothesis
HFG	Humanforschungsgesetz (Law on human research)
HMG	Heilmittelgesetz
HRA	Federal Act on Research involving Human Beings
IMP	Investigational Medicinal Product
IIT	Investigator-initiated Trial
ISO	International Organisation for Standardisation
ITT	Intention to treat
KlinV	Verordnung über klinische Versuche in der Humanforschung *(in English: ClinO, in French OClin)*
LPTh	Loi sur les produits thérapeutiques
LRH	Loi fédérale relative à la recherche sur l'être humain
MD	Medical Device
OClin	Ordonnance sur les essais cliniques dans le cadre de la recherche sur l'être humain *(in German : KlinV, in English : ClinO)*
PI	Principal Investigator
SDV	Source Data Verification
SOP	Standard Operating Procedure
SPC	Summary of product characteristics
SUSAR	Suspected Unexpected Serious Adverse Reaction
TMF	Trial Master File

STUDY SCHEDULE

Study schedule

Time point<br>weeks post-transplant	0	<2	...	4	5	6	...	10	11	12	...	20	22	24	...	52
**Study inclusion procedure**																
Study eligibility		X														
Randomization		X														
Demographics		X														
Medical history		X														
**Continuous assessment of routine and study parameters**																
Assessment of events (AE, SAE, rejection etc.)	anytime															
Routine clinical and laboratory assessment		X		X	X	X	X	X	X	X	X	X	X	X	X	X
Urine sediment +/- culture		X		X	X	X	X	X	X	X	X	X	X	X	X	X
Decoy cells				X	X	X	X	X	X	X	X	X	X	X		X
Study parameters (urine CXCL10/creatinine)				X	X	X		X	X	X		X	X	X		X
**Allograft biopsies**																
- Implantation biopsy	X															
- Clinically indicated*	anytime as per treating physician															
- CXCL10-triggered (only interventional arm)*						X<br>if 4+5w CXCL10 high in the absence of UTI/BKV				X<br>if 10+11w CXCL10 high in the absence of UTI/BKV				X<br>if 20+22w CXCL10 high in the absence of UTI/BKV		
- One-year readout biopsy																X

*Additional urine collections 2 and 4 weeks after treated rejection for CXCL10 measurements

1. STUDY ADMINISTRATIVE STRUCTURE

1.1 Sponsor, Sponsor-Investigator

University Hospital Basel,

Represented by the PI Prof. Stefan Schaub
Transplantation Immunology and Nephrology
University Hospital Basel
Petersgraben 4
4031 Basel
Email: stefan.schaub@usb.ch
Phone: 061 265 45 33

Funded by the SNF (grant 32003B_169310 / 1).

1.2 Principal Investigator(s)

Prof. Stefan Schaub
Transplantation Immunology and Nephrology
University Hospital Basel
Petersgraben 4
4031 Basel

Email: stefan.schaub@usb.ch

Phone: 061 265 45 33

PD Dr. Patricia Hirt-Minkowski
Transplantation Immunology and Nephrology
University Hospital Basel
Petersgraben 4
4031 Basel
Email: patricia.hirt-minkowski@usb.ch
Phone: 061 556 56 22

PD Dr. Helmut Hopfer
Institute of Pathology
University Hospital Basel
Petersgraben 4
4031 Basel
Email: helmut.hopfer@usb.ch
Phone: 061 328 78 90

PD Dr. Axel Regeniter
Department of Laboratory Medicine
University Hospital Basel
Petersgraben 4
4031 Basel
Email: axel.regeniter@usb.ch

Phone: 061 328 62 29

1.3 Statistician ("Biostatistician")

PD Dr. Michael Koller

Director STCS Data center

Email: michael.koller@usb.ch

1.4 Laboratory

General lab work:

PD Dr. Axel Regeniter
Department of Laboratory Medicine
University Hospital Basel
Petersgraben 4
4031 Basel
Email: axel.regeniter@usb.ch
Phone: 061 328 62 29

Chemokine measurements will be performed at the Department of Biomedicine by a dedicated and trained person, who is part of the study team.

1.5 Monitoring institution

None.

1.6 Data Safety Monitoring Committee

Prof. Jürg Steiger
Transplantation Immunology and Nephrology
University Hospital Basel
Petersgraben 4
4031 Basel
Email: juerg.steiger@usb.ch
Phone: 061 328 64 06

Prof. Michael Dickenmann
Transplantation Immunology and Nephrology
University Hospital Basel
Petersgraben 4
4031 Basel
Email: michael.dickenmann@usb.ch
Phone: 061 328 64 39

PD Dr. Michael Koller
Director STCS Data center
Email: michael.koller@usb.ch

1.7 Any other relevant Committee, Person, Organisation, Institution

Not applicable.

2. ETHICAL AND REGULATORY ASPECTS

The decision of the CEC and Swissmedic/foreign competent authority concerning the conduct of the study will be made in writing to the Sponsor-Investigator before commencement of this study. The clinical study can only begin once approval from all required authorities has been received. Any additional requirements imposed by the authorities shall be implemented.

2.1 Study registration

The study will be registered according to existing regulations at clinicaltrials.gov and in the Swiss Federal Complementary Database.

2.2 Categorisation of study

After consultation with the scientific secretary of the EKNZ the study falls into “Other clinical trials”, category B (according to the HFG Art. 60).

2.3 Competent Ethics Committee (CEC)

The responsible investigator ensures that approval from an appropriately constituted Competent Ethics Committee (CEC) is sought for the clinical study. No changes are made to the protocol without prior Sponsor and CEC approval, except where necessary to eliminate apparent immediate hazards to study participants.

Premature study end or interruption of the study is reported within 15 days. The regular end of the study is reported to the CEC within 90 days, the final study report shall be submitted within one year after study end. Amendments are reported according to chapter 2.10.

2.4 Competent Authorities (CA)

Due to the categorization as “Other clinical trials” no approval is required from CA.

2.5 Ethical Conduct of the Study

The study will be carried out in accordance to the protocol and with principles enunciated in the current version of the Declaration of Helsinki, the guidelines of Good Clinical Practice (GCP) issued by ICH, in case of medical device: the European Directive on medical devices 93/42/EEC and the ISO Norm 14155 and ISO 14971, the Swiss Law and Swiss regulatory authority’s requirements. The CEC and regulatory authorities will receive annual safety and interim reports and be informed about study stop/end in agreement with local requirements.

2.6 Declaration of interest

No conflict of interest to declare.

2.7 Patient Information and Informed Consent

e.g. The investigators will explain to each participant the nature of the study, its purpose, the procedures involved, the expected duration, the potential risks and benefits and any discomfort it may entail. Each participant will be informed that the participation in the study is voluntary and that he/she may withdraw from the study at any time and that withdrawal of consent will not affect his/her subsequent medical assistance and treatment.

The participant must be informed that his/her medical records may be examined by authorised individuals other than their treating physician.

All participants for the study will be provided a participant information sheet and a consent form describing the study and providing sufficient information for participant to make an informed decision about their participation in the study. The participant has several days to decide whether he/she wants to participate.

The patient information sheet and the consent form will be submitted to the CEC and to the competent authority (as applicable) to be reviewed and approved. The formal consent of a participant, using the approved consent form, must be obtained before the participant is submitted to any study procedure.

The participant should read and consider the statement before signing and dating the informed consent form, and should be given a copy of the signed document. The consent form must also be signed and dated by the investigator (or his designee) and it will be retained as part of the study

records.

2.8 Participant privacy and confidentiality

The investigator affirms and upholds the principle of the participant's right to privacy and that they shall comply with applicable privacy laws. Especially, anonymity of the participants shall be guaranteed when presenting the data at scientific meetings or publishing them in scientific journals.

Individual subject medical information obtained as a result of this study is considered confidential and disclosure to third parties is prohibited. Subject confidentiality will be further ensured by utilising subject identification code numbers to correspond to treatment data in the computer files.

For data verification purposes, authorised representatives of the Sponsor (-Investigator), a competent authority (e.g. Swissmedic), or an ethics committee may require direct access to parts of the medical records relevant to the study, including participants' medical history.

2.9 Early termination of the study

The Sponsor-Investigator may terminate the study prematurely according to certain circumstances, for example:

- ethical concerns,
- insufficient participant recruitment,
- when the safety of the participants is doubtful or at risk, respectively,
- alterations in accepted clinical practice that make the continuation of a clinical trial unwise,
- early evidence of benefit or harm of the experimental intervention

See also 11.3 statistical criteria of termination of trial.

2.10 Protocol amendments

Substantial amendments are only implemented after approval of the CEC.

Under emergency circumstances, deviations from the protocol to protect the rights, safety and well-being of human subjects may proceed without prior approval of the sponsor and the CEC. Such deviations shall be documented and reported to the sponsor and the CEC as soon as possible.

All Non-substantial amendments are communicated to the CEC as soon as possible.

3. BACKGROUND AND RATIONALE

3.1 Background and Rationale

3.1.1) Rejection is the leading cause of death-censored renal allograft failure

Renal replacement therapy due to end-stage renal disease is common (~3700 Swiss are currently on dialysis) and costly (~100'000 CHF/patient/year). Transplantation is the therapy of choice for many patients due to improved survival, better quality of life and it has significant cost-savings after the first year post-transplant compared to dialysis (~80'000 CHF/patient/year).

A major challenge in transplantation is how to optimize anti-rejection therapy to balance the risk of rejection from under-immunosuppression against the risk of infections/cancer from over-immunosuppression. The ideal regimen would provide the minimum therapy to avoid complications while being sufficient to prevent rejection. Recent studies found that allograft rejection is the leading cause for death-censored allograft failure (30-40%) (1,2). This is in line with data from our center, where 56% of death-censored allograft failures occurring from 2005 to 2015 were related to rejection (3).

3.1.2) Current concept of rejection events leading to allograft failure

In patients without pre-formed donor-specific memory (i.e. absence of donor-specific HLA-antibodies [HLA-DSA]), T-cell mediated rejection (TCMR) is the most common rejection phenotype. Using modern immunosuppression, TCMR mainly presents as a smoldering subclinical process. Indeed, the one-year incidence of renal allograft rejection with functional decline measured by serum creatinine (i.e. clinical rejection) is around 10-15% (4), while additional 10-30% experience rejection with stable creatinine (i.e. subclinical rejection) (5-8).

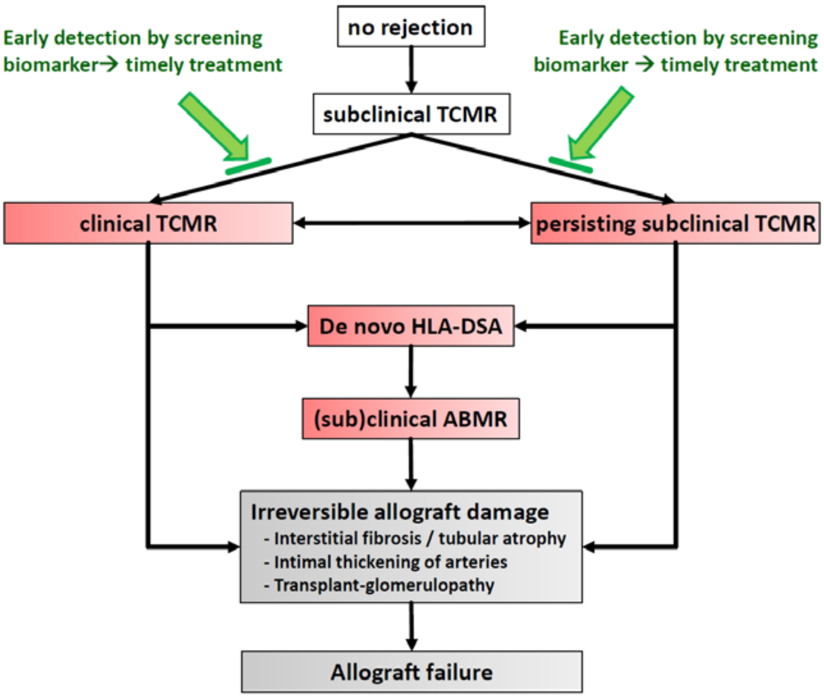

Figure 1: Current concept of rejection events leading to allograft failure and the stage at which a biomarker-based intervention would intervene.

Persisting subclinical TCMR and/or inadequately treated clinical TCMR can both lead directly or via induction of *de novo* HLA-DSA to irreversible allograft damage and ultimately allograft failure (5,9-16) (***Figure 1***). Thus, timely intervention at the level of subclinical TCMR might improve allograft outcomes. In fact, treatment of subclinical TCMR in patients on cyclosporine-based therapy in two randomized, controlled trials lead to diminished histological injury and improved functional outcomes (17,18). In addition, treating subclinical TCMR in patients on modern immunosuppression resulted in similar long-term graft survival compared to those without rejection (12). Altogether, these data show that subclinical TCMR is clinically significant and that early effective therapy can improve long-term outcomes.

3.1.3) Biomarker-based approach for early detection of TCMR

The key question is how to detect TCMR at an early, still subclinical stage allowing for timely therapeutic interventions.

One option is to perform regular surveillance biopsies. Most centers using surveillance biopsies for monitoring perform one or two biopsies within the first year post-transplant (e.g. at month 3, month 6, or at one year). This strategy has well-known limitations. First, the rejection process is dynamic and

can occur at any time post-transplant. Therefore, the surveillance biopsy grid (e.g. month 6 and one year) is not frequent enough to detect subclinical TCMR at an early stage. Second, about 2/3 of all surveillance biopsies do not show any rejection and would thus not have been necessary to guide the immunosuppressive therapy (19). Skipping these less informative biopsies would lower the costs and reduce the inconvenience for patients associated with the invasive biopsy procedure.

Another option is to guide the performance of surveillance biopsies by using a non-invasive screening biomarker. The advantage of such a strategy is that regular testing can be performed at much more frequent intervals (e.g. weekly/bi-weekly/monthly). This offers the opportunity to detect TCMR earlier than with scheduled surveillance biopsies and/or before the TCMR becomes clinically apparent indicated by an elevation of serum creatinine. Therefore, the intervention can be initiated even earlier than with scheduled surveillance biopsies and might further improve allograft outcomes (***Figure 1***). Moreover, this strategy might substantially reduce the number of biopsies compared to a scheduled surveillance biopsy protocol (19).

Many biomarker discovery studies have been performed in the last ten years using different sources (e.g. serum, PBMC's, urine) and different technologies (e.g. ELISA, ELISPOT, PCR, proteomics & genomics) (20,21). The urine CXCL10 chemokine has been consistently found to be associated with subclinical allograft rejection and it rises before rejection becomes clinically apparent (i.e. elevation of serum creatinine) (19,22-30). Not surprising, a recent JASN editorial states "sufficient information exists that CXCL10 is associated with ongoing acute rejection in the kidney. We think it is time to put diagnostic +/- prognostic biomarkers to direct testing." (31).

3.1.4) Urine CXCL10 as a biomarker to detect allograft rejection and predict long-term outcomes

Since 2004, we collaborate with the Winnipeg Transplant Group (Dr. J. Ho, Dr. P. Nickerson, and Dr. D. Rush) to discover and validate novel biomarker for non-invasive monitoring of renal allograft recipients. This research group has made major contributions in the development of urine CXCL10 as a biomarker (19,22-25).

Our group was the first to show that urine CXCL10 can detect subclinical TCMR (23). We were also the first to validate these results in an unselected real-life population allowing calculation of reliable diagnostic characteristics (19). ***Figure 2*** summarizes urine CXCL10 results for subclinical pathologies (362 surveillance biopsies from 213 consecutive patients in Basel). Notably, in this study the histological groups were equal with regard to eGFR and proteinuria indicating that these standard monitoring parameters were not able to separate them. By contrast, urine CXCL10 increases in a stepwise manner with the extent of tubulointerstitial inflammation. The calculated CXCL10/creat cut-off to separate 'no rejection' vs. 'subclinical rejection' was 1.535ng/mmol (AUC 0.69, sensitivity 61%, specificity 72%).

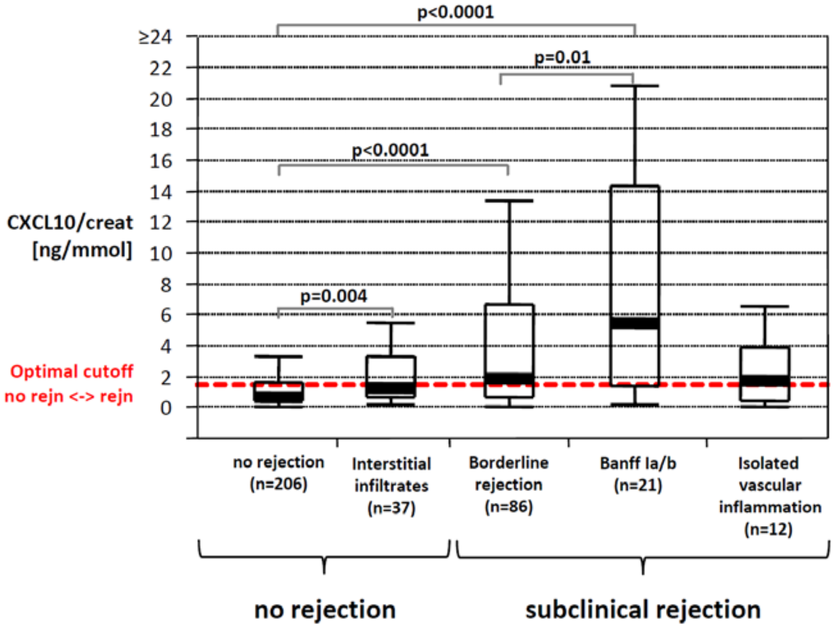

Figure 2: Urine CXCL10/creatinine levels in 362 surveillance biopsies

Furthermore, our group was the first to demonstrate that urine CXCL10 has prognostic value for prediction of long-term graft outcomes (i.e. composite endpoint consisting of death-censored graft loss, late biopsy-proven rejection and deterioration of allograft function) (***Figure 3, next page***) (22). Only 2/62 patients (3%) with low 6-month CXCL10 levels (<0.70 ng/mmol) experienced late rejection or graft loss due to rejection compared to 15/92 patients (16%) with high 6-month CXCL10 levels (p=0.008) (22).

Moreover, our group has also explored the urine CCL2 chemokine as a prognostic biomarker, which provides incremental value in addition to CXCL10 for prediction of intermediate and long-term outcomes (32-35). We regard urine CXCL10 as a biomarker for allograft inflammation, while urine CCL2 reflects progression of inflammation into irreversible fibrosis.

In summary, our work demonstrates that urine CXCL10 is a promising biomarker to detect subclinical rejection exceeding the current clinical standard-of-care monitoring (i.e. serum creatinine and proteinuria). In addition, both urine CXCL10 and CCL2 have prognostic value to predict long-term outcomes enabling tailored patient monitoring and immunosuppression.

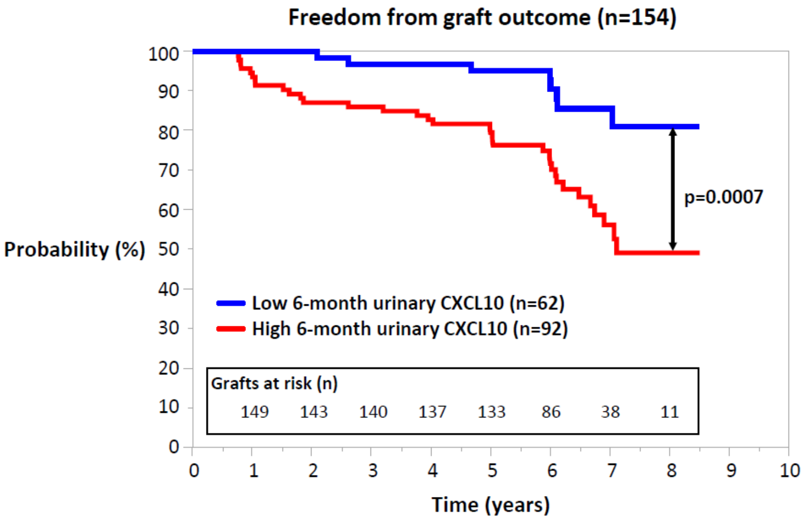

Figure 3: 6-month urine CXCL10/creatinine levels and long-term graft outcome

<u>3.1.5) Establishing an analytical platform and defining a CXCL10 cutoff to trigger investigations</u>

In all our mentioned retrospective studies, CXCL10 measurements were performed by a custom ELISA, which was very robust but time-consuming (4 day procedure). For a prospective interventional study, a sensitive, robust and scalable platform with a rapid turnaround time is of major importance. After evaluation of different platforms/assays, we decided to use the MesoScale instrument and the VPlex assays (www.mesoscale.com).

The reasons are:

- Rapid results within one day
- Highly sensitive
- Robust (intra-assay CV 3.7%, inter-assay CV 7.6%)
- Monoclonal antibody detection system
- ISO certified organization with inter-assay CV's that meet FDA criteria for a quantitative assay
- Experience in translating research assays to clinically available ones

After optimization of the MesoScale assay, we reanalysed all urines from the previous study, where the diagnostic criteria of urine CXCL10 for detection of subclinical rejection were derived from (19) (see also ***Figure 2***). The results could be reproduced using the MesoScale platform and the correlation with the ELISA values were very good (r^2=0.93). The established CXCL10/creatinine cut-off with the MesoScale instrument to separate 'no rejection' vs 'subclinical rejection' was 0.95ng/mmol. Including a CV of 5%, we decided to use a cutoff of 1.00ng/mmol for this study.

3.2 Investigational Product (treatment, device) and Indication

This study investigates whether early treatment of rejection, as detected by urinary CXCL10, improves transplant outcomes. There is no investigational treatment, because the used drugs for treatment of rejection are identical between the two groups.

3.3 Preclinical Evidence

Uptodate, there are no available data from randomized trials because this study is the first of its kind.

3.4 Clinical Evidence to Date

Uptodate, there are no available data from randomized trials because this study is the first of its kind.

3.5 Dose Rationale / Medical Device: Rationale for the intended purpose in study (pre-market MD)

Not applicable.

3.6 Explanation for choice of comparator (or placebo)

The comparator for this study is the current standard-of-care monitoring for renal allograft recipients relying on serum creatinine measurements.

3.7 Risks / Benefits

The control arm of the study consists of the current standard-of-care in most transplant centres worldwide. The interventional arm will very likely lead to more allograft biopsies, which itself carries a small risk of complications. In our centre, the most relevant complication is bleeding, which occurs in less than 1% of interventions (bleeding requiring a blood transfusion occurs in 0.1%). Furthermore, patients randomized into the interventional arm will likely receive more anti-rejection treatment with better control of rejection-related damage to the allograft, but with an increased risk of immunosuppression-associated side effects. Overall, the benefit of an early individualized treatment strategy to prevent ongoing allograft rejection is larger than the anticipated risks.

3.8 Justification of choice of study population

The study population essentially consists of unselected consecutive renal allograft recipients, because…

(i) the investigated approach should eventually be implemented in this population.
(ii) all prior study results use for the design of this prospective study were derived from an unselected consecutive patient population.

4. STUDY OBJECTIVES

4.1 Overall Objective

In this proposal we will test the hypothesis that early treatment of rejection detected by urine CXCL10 monitoring will improve graft outcomes compared to standard monitoring by serum creatinine.

4.2 Primary Objective

To determine the effectiveness of early treatment of rejection, as detected by urine CXCL10, to improve graft outcomes.

4.3 Secondary Objectives

- To investigate the urine CXCL10 kinetics in response to anti-rejection therapy.
- To evaluate and independently validate different novel diagnostic and prognostic markers for rejection or long-term outcomes (exploratory objective).

4.4 Safety Objectives

To investigate the safety of the approach mentioned in 5.2 (i.e. biopsy-related complications and immunosuppression-related complications [infections, malignancies])

5. STUDY OUTCOMES

5.1 Primary Outcome

A major barrier in transplant trials is that FDA-accepted outcomes (1-year graft loss and clinical rejection) are no longer sufficient to assess interventions aimed at improving long-term allograft survival in this era of modern immunosuppression (36,37). Histopathology surrogates under consideration for FDA-approval will be used in our composite outcome. The trial will be registered and long-term follow-up obtained for hard outcomes (eg. graft loss) after the 1st year. **The primary 1-year composite outcome will consist of at least one of the following four outcomes:**

- **Graft loss not due to death of the patient** (anticipated incidence 2%)
- **Biopsy-proven clinical acute rejection from 4-weeks to 1-year post-transplant** (anticipated incidence 13-14% (8)), classified according to Banff criteria (38). If serum creatinine rises after elevated urine CXCL10, but prior to study biopsy (eg. pending repeat urine/biopsy booking), these will not be considered clinical rejection as it was first detected by CXCL10.
- **Subclinical T-cell mediated rejection in 1-year surveillance biopsy** (anticipated incidence 15%), classified according to Banff criteria and defined by t>0 and/or v>0. As in our population the incidence of subclinical TCMR at 6 months is 20-25% (8,39) [reference 39 refers to a manuscript in preparation], we conservatively assume an incidence of 15% at 1 year. Patients with tubulitis (t>0) at 1 year have an increased risk of graft loss compared to those with minor histological change (40). Indeed, late subclinical rejection has been shown to result in worsening IFTA, declining graft function and graft loss (7,41).
- **Interstitial fibrosis / tubular atrophy with inflammation (IFTA+I defined by the Mayo Clinic criteria) in 1-year surveillance biopsy** (anticipated incidence 9.5% (10,42)). IFTA+i is a strong marker for graft loss (10,40-43) and mild forms where the degree of inflammation does not meet diagnostic criteria for borderline rejection are strongly and independently associated with functional decline and graft loss (10,42,43). It is associated with prior acute rejection, increased HLA mismatch (10), and a rejection gene signature (42). This body of work strongly suggests that IFTA+i reflects an ongoing, low-grade T-cell mediated rejection state that is not recognized in the current Banff schema (10).

5.2 Secondary Outcomes

Efficacy: Microvascular inflammation at 1-year (ptc, g, c4d, cg); Development of IFTA from implantation to 1-year (Δ ci, ct, cv); Days from transplantation to biopsy-proven clinical acute rejection; Proteinuria >500mg/day at 6- and 12-months post-transplant

Long-term outcomes: Graft loss including its cause, death including its cause, allograft function (creatinine and eGFR), proteinuria, biopsy-proven rejection

5.3 Other Outcomes of Interest

Correlation of histological evolution of rejection with the evolution of CXCL10 chemokine levels.

It is currently unknown, how CXCL10 chemokine levels change in response to rejection treatment and if these changes correspond to the histological evolution. If we can demonstrate that CXCL10 changes correlate with histological evolution, we can use urine CXCL10 to monitor the response to rejection treatment in the future.

To evaluate and independently validate different novel diagnostic and prognostic markers for rejection or long-term outcomes.

We and others have shown that urine chemokines are predictors for rejection (CXCL10) as well as long-term outcomes (CXCL10 and CCL2) in several retrospective cohorts. A validation of these results in a prospective study is important.

In addition, if the corresponding consent form "**Einwilligungserklärung für Weiterverwendung von biologischem Material**" if signed, novel biomarker might be explored in the available biobanked samples.

5.4 Safety Outcomes

The interventional arm will very likely have more allograft biopsies and more rejection treatments. Therefore, the safety outcomes are:

- Number of total, indication and CXCL10-triggered biopsies within the first year post-transplant
- Biopsy-related complications (bleeding requiring hospitalization, blood transfusion or other interventions) within the first year post-transplant
- Immunosuppression-related complications (infections, malignancies) within the first year post-transplant

6. STUDY DESIGN

6.1 General study design and justification of design

This is a single-center randomized controlled, two parallel arm, unblinded clinical effectiveness trial. The follow-up for the primary 1-year composite outcome is one year. Long-term follow-up for pertinent outcomes (allograft survival, patient survival, allograft function) will be collected, but the patients do not have any study-specific assessments and/or interventions beyond the first year post-transplant. The study design is detailed in the following Figure.

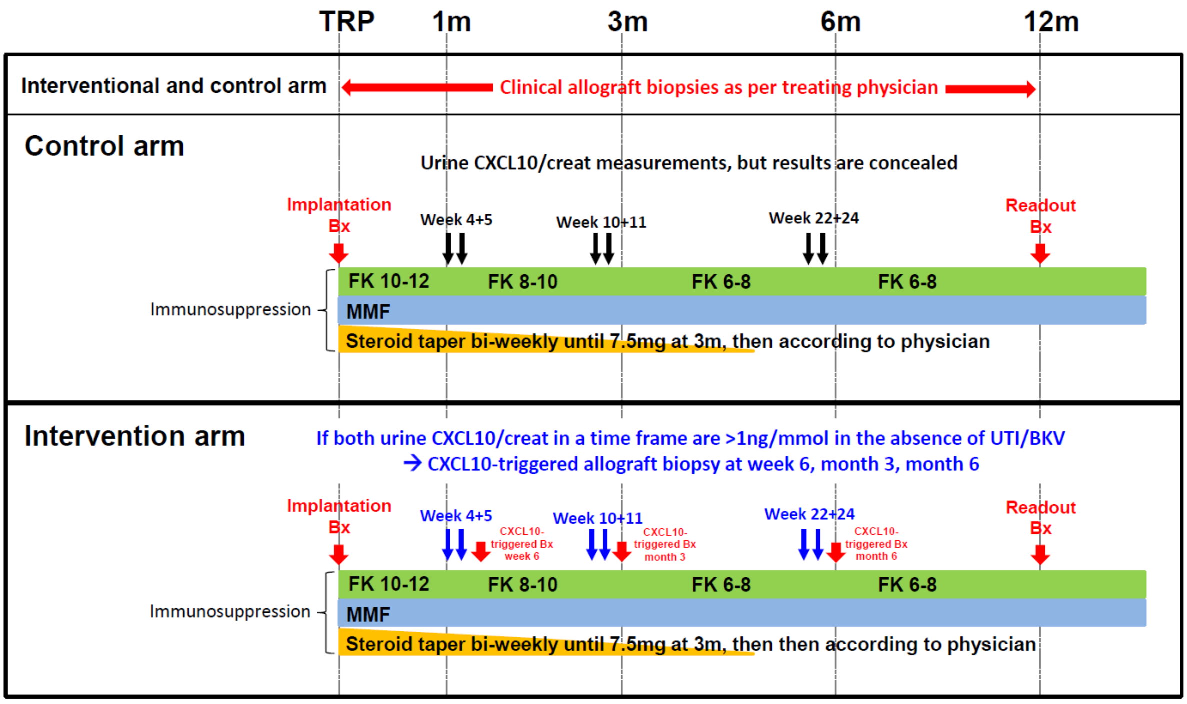

Measurements of urine CXCL10 in the control arm are important for two reasons. First, it will allow to demonstrate potential CXCL10 differences between the two groups. Second, it will provide data whether clinical rejection would have been anticipated by preceding elevated CXCL10 levels.

The study has very broad inclusion criteria making around 95% of all patients receiving a renal allograft at our centre eligible for inclusion. We define at our centre two distinct immunological risk profiles, which are used to select the immunosuppressive regimen. In order to be able to investigate the study intervention in these distinct immunological risk groups separately and to reduce the bias of the immunosuppressive regimen, we will stratify the transplants by their immunological risk. These two distinct groups will be randomized and analysed separately. The two immunological risk profiles are:

- Normal risk transplants (i.e. no immunological risk as detailed below)
- High risk transplants defined as
    - ABO-incompatible and/or
    - presence of donor-specific HLA-antibodies and/or
    - husband-to-wife transplant with shared children or child-to-mother transplant

6.2 Methods of minimising bias

6.2.1 Randomisation

We will use computer-generated randomization, stratified by the two distinct immunological risk profiles with an overall 1:1 allocation. The randomization list will be implemented into the study database. Patient recruitment and randomization will be performed by the study team.

6.2.2 Blinding procedures

Due to the biopsy/interventional nature of this trial, randomization cannot be blinded to the

investigators or participants. The 1-yr surveillance biopsies (read-out biopsy) will be read and scored by a local nephropathologist for clinical purposes. A second local nephropathologist, who is blinded to the study arm and the results of prior allograft biopsies of the participants, will re-read and re-score the 1-yr surveillance biopsies for this study.

6.2.3 Other methods of minimising bias

Not applicable.

6.3 Unblinding Procedures (Code break)

Not applicable.

7. STUDY POPULATION

7.1 Eligibility criteria

Participants fulfilling all of the following inclusion criteria are eligible for the study:

- Informed Consent as documented by signature
- Adult recipient of a renal allograft at the University Hospital Basel

The presence of any one of the following exclusion criteria will lead to exclusion of the participant:

- HLA-identical living donor transplantation
- Primary non-function
- Participation in immunosuppression interventional trials

Current pregnancy at the time of scheduled renal transplantation is a general contraindication for renal transplantation.

7.2 Recruitment and screening

The study team members and the responsible physicians at the Clinic for Transplantation Immunology & Nephrology will recruit the patients. They will explain and discuss the Informed Consent Form in detail. Recruitment will be performed within the first two weeks after transplantation. The study participants will not receive any payment or compensation.

7.3 Assignment to study groups

If the patient has signed the Informed Consent Form, a study team member or the responsible physician at the Clinic for Transplantation Immunology & Nephrology will use the computer-generated randomization list to assign the study arm.

7.4 Criteria for withdrawal / discontinuation of participants

Withdrawal of informed consent can be done at any time without giving any specific reasons. Withdrawal will be noted in the patient file. Potential reasons for patient withdrawal from the study are: (1) incompliance with the study protocol, (2) patient is moving away, (3) wish of the patient

8. STUDY INTERVENTION

8.1 Identity of Investigational Products (treatment / medical device)

The study design was described in 6.1.

8.1.1 Experimental Intervention (treatment / medical device)

In the interventional arm, urine CXCL10 chemokine monitoring will be used to trigger performance of allograft biopsies. Urine CXCL10 measurements will be performed with a Meso QuickPlex SQ120 instrument using V-PLEX plates.

8.1.2 Control Intervention (standard/routine/comparator treatment / medical device)

The control group has clinical standard-of-care monitoring.

8.1.3 Packaging, Labelling and Supply (re-supply)

Not applicable.

8.1.4 Storage Conditions

Not applicable.

8.2 Administration of experimental and control interventions

8.2.1 Experimental Intervention

Not applicable.

8.2.2 Control Intervention

Not applicable.

8.3 Dose / Device modifications

Not applicable.

8.4 Compliance with study intervention

Our renal allograft recipients have in general an extremely high adherence to medication, procedures and interventions. We do not expect any problems in this regard.

8.5 Data Collection and Follow-up for withdrawn participants

We expect a very low rate of withdrawals. If so, we will only collect those data which we obtain by clinical standard-of-care. Long-term follow-up is guaranteed and ensured by existing regulations.

8.6 Trial specific preventive measures

Not applicable.

8.7 Concomitant Interventions (treatments)

Concomitant immunosuppression trials are prohibited at the inclusion step. Other interventions without any influence on the study interpretation are allowed.

8.8 Study Drug / Medical Device Accountability

Not applicable.

8.9 Return or Destruction of Study Drug / Medical Device

Not applicable.

9. STUDY ASSESSMENTS

9.1 Study flow chart(s) / table of study procedures and assessments

The allowed time frames for the visits are dependent on the time post-transplant. For the first 12 weeks, the time frames are +/-7 days, thereafter the time frames are +/- 14 days.

Study schedule

Time point weeks post-transplant	0	<2	...	4	5	6	...	10	11	12	...	20	22	24	...	52
**Study inclusion procedure**																
Study eligibility		X														
Randomization		X														
Demographics		X														
Medical history		X														
**Continuous assessment of routine and study parameters**																
Assessment of events (AE, SAE, rejection etc.)	anytime															
Routine clinical and laboratory assessment		X		X	X	X	X	X	X	X	X	X	X	X	X	X
Urine sediment +/- culture		X		X	X	X	X	X	X	X	X	X	X	X	X	X
Decoy cells				X	X	X	X	X	X	X	X	X	X	X		X
Study parameters (urine CXCL10/creatinine)				X	X	X		X	X	X		X	X	X		X
**Allograft biopsies**																
- Implantation biopsy	X															
- Clinically indicated*	anytime as per treating physician															
- CXCL10-triggered (only interventional arm)*						X if 4+5w CXCL10 high in the absence of UTI/BKV				X if 10+11w CXCL10 high in the absence of UTI/BKV				X if 20+22w CXCL10 high in the absence of UTI/BKV		
- One-year readout biopsy																X

*Additional urine collections 2 and 4 weeks after treated rejection for CXCL10 measurements

9.2 Assessments of outcomes

9.2.1 Assessment of primary outcome

The primary 1-year composite outcome will consist of at least one of the following four outcomes:

- Graft loss not due to death of the patient, or
- Biopsy-proven clinical acute rejection from 4-weeks to 1-year post-transplant, or
- Subclinical T-cell mediated rejection in 1-year surveillance biopsy defined by t>0 and/or v>0, or
- Interstitial fibrosis / tubular atrophy with inflammation (IFTA+i defined by the Mayo Clinic criteria) in 1-year surveillance biopsy

9.2.2 Assessment of secondary outcomes

Efficacy:

- Microvascular inflammation at 1-year (ptc, g, c4d, cg), classified according to Banff criteria (38)
- Development of IFTA from implantation to 1-year (∆ ci, ct, cv), classified according to Banff criteria (38)
- Days from transplantation to biopsy-proven clinical acute rejection
- Proteinuria >500mg/day at 6- and 12-months post-transplant

Safety:

- Number of total, indication and CXCL10-triggered biopsies within the first year post-transplant
- Biopsy-related complications (e.g. hematuria requiring intervention, prolonged hospitalization) within the first year post-transplant
- immunosuppression-related complications (infections, malignancies) within the first year post-transplant

Long-term outcomes:

- Graft loss including its cause
- Death including its cause
- Allograft function (creatinine and eGFR)
- Proteinuria
- Biopsy-proven rejection

9.2.3 Assessment of other outcomes of interest

Urine CXCL10 kinetics in response to anti-rejection therapy:

Urine CXCL10 will be measured according to our SOP. Serial histology will be classified according to Banff criteria (38)

Evaluation and validatation of different novel diagnostic and prognostic markers for rejection or long-term outcomes:

Rejection will be classified according to Banff criteria (38), short and long-term outcomes include eGFR, allograft loss as well as death

9.2.4 Assessment of safety outcomes

Adverse events clearly due to the interventional arm are restricted to biopsy-related complications and immunosuppression-related complications.

9.2.4.1 Adverse events

Biopsy-related complications and immunosuppression-related complications are secondary outcomes and are documented and collected.

9.2.4.2 Laboratory parameters

Not applicable.

9.2.4.3 Vital signs

Not applicable.

9.2.5 Assessments in participants who prematurely stop the study

Participants who prematurely stop the study we be followed by standard-of-care monitoring used in our clinic.

9.3 Procedures at each visit

All patients in this study will be monitored by standard-of-care clinical practice in our clinic detailed in 9.1. Patients in the study will have additional tests (i.e. urine CXCL10 measurments) at specific time points. The patients do NOT have additional visits compared to patients not included in this study.

However, patients in the intervention arm may have up to three CXCL10-triggered allograft biopsies. These will be performed as any allograft biopsy according to clinical standard-of-care.

Adjustments of the immunosuppression in both arms are based on the time post-transplant, allograft biopsy results, the clinical course and immunosuppression-related side effects.

Maintenance Immunosuppression and Treatment of Rejection

Time point<br>months post-transplant	1	2	3	4	5	6	7	8	9	10	11	12

Normal risk transplants (at least one HLA-mismatch and not high risk transplant)

Immunosuppression in the absence of rejection

Basiliximab (day 0 and 4)

	1	2	3	4	5	6	7	8	9	10	11	12
Tacrolismus (trough level)	10-12	8-10		6-8								
MPA (trough level)	>2											
Steroids	Steroid taper bi-weekly until 7.5mg at 3 mt, then at discretion of physician											

High risk transplants (ABO-incompatible or HLA-DSA or husband-to-wife with shared children or child-to-mother)

Immunosuppression in the absence of rejection

Induction according to specific protocols)*

	1	2	3	4	5	6	7	8	9	10	11	12
Tacrolismus (trough level)	10-12	8-10		6-8								
MPA (trough level)	>2											
Steroids	Steroid taper bi-weekly until 7.5mg at 3 mt, then continue at 0.1mg/kg KG. Withdrawal at discretion of physician.											

* Induction protocols
ABOi: Rituximab +/- immunoabsorption
HLA-DSA: ATG + IvIg
Husband-to-wife with shared children but no HLA-DSA: ATG

Treatment of clinical rejection (general recommendations)	
- Borderline TCMR	Steroids p.o or i.v. with taper
- TCMR IA-IB	Steroids i.v. 3-5x with taper
- TCMR ≥IIA	Steroids i.v. 3-5x or ATG with steroid taper
- ABMR	Steroids i.v. plus ATG plus IvIg plus PP depending on the context and severity

Treatment of subclinical rejection (general recommendations)	
- Borderline TCMR	Steroids p.o with taper
- TCMR IA-IB	Steroids p.o. or i.v. 3-5x with taper
- TCMR ≥IIA	Steroids p.o. or i.v. 3-5x or ATG depending on the context and severity with steroid taper
- ABMR	Steroids p.o., steroids i.v., or ATG depending on the context and severity

9.3.1 Study inclusion visit

This will be conducted during the first 14 days post-transplant (mainly during the hospitalization). Patients will be screened for study inclusion. If they provide informed consent, baseline data will be obtained by chart review.

9.3.2 Regular visits until the end of the first year post-transplant

As detailed above, all visits are regular visits. Only in patients allocated to the intervention arm, additional visits for allograft biopsies may be scheduled depending on the urine CXCL10 results.

9.3.3 One-year study visit including read-out allograft biopsy

During this visit standard-of-care parameters will be obtained. In addition, all patients will have a read-out allograft biopsy.

9.3.4 Long-term follow-up

All study interventions and study visits cease at 1 year post-transplant. Abbreviated patients follow-up in all enrolled patients will be performed to obtain pertinent long-term follow-up beyond the first year post-transplant. These data will be collected in the context of the annual patient follow-up.

10. SAFETY

Neither the drug studies (10.1) nor the device studies (10.2) sections are entirely appropriate for this study. We will use the 10.1. section as a template. The investigated safety outcomes have been described in 9.2.2 and repeated here:

- Number of total, indication and CXCL10-triggered biopsies within the first year post-transplant
- Biopsy-related complications (e.g. hematuria requiring intervention, prolonged hospitalization) within the first year post-transplant
- Immunosuppression-related complications (infections, malignancies) within the first year post-transplant

10.1 Drug studies

10.1.1 Definition and assessment of (serious) adverse events and other safety related events

An **Adverse Event (AE)** is any untoward medical occurrence in a patient or a clinical investigation participant administered a pharmaceutical product and which does not necessarily have a causal relationship with the study procedure. An AE can therefore be any unfavourable and unintended sign (including an abnormal laboratory finding), symptom, or disease temporally associated with the use of a medicinal (investigational) product, whether or not related to the medicinal (investigational) product. [ICH E6 1.2]

A **Serious Adverse Event (SAE)** is classified as any untoward medical occurrence that:

- results in death,
- is life-threatening,
- requires in-patient hospitalization or prolongation of existing hospitalisation,
- results in persistent or significant disability/incapacity, or
- is a congenital anomaly/birth defect.

In addition, important medical events that may not be immediately life-threatening or result in death, or require hospitalisation, but may jeopardise the patient or may require intervention to prevent one of the other outcomes listed above should also usually be considered serious. [ICH E2A]

SAEs should be followed until resolution or stabilisation. Participants with ongoing SAEs at study termination (including safety visit) will be further followed up until recovery or until stabilisation of the disease after termination.

Assessment of Causality

Both Investigator and Sponsor-investigator make a causality assessment of the event to the study drug, based on the criteria listed in the ICH E2A guidelines:

Relationship	Description
Definitely	Temporal relationship<br>Improvement after dechallenge*<br>Recurrence after rechallenge<br>(or other proof of drug cause)
Probably	Temporal relationship<br>Improvement after dechallenge

	No other cause evident
Possibly	Temporal relationship<br>Other cause possible
Unlikely	Any assessable reaction that does not fulfil the above conditions
Not related	Causal relationship can be ruled out
*Improvement after dechallenge only taken into consideration, if applicable to reaction	

Unexpected Adverse Drug Reaction

An "unexpected" adverse drug reaction is an adverse reaction, the nature or severity of which is not consistent with the applicable product information (e.g. Investigator's Brochure for drugs that are not yet approved and Product Information for approved drugs, respectively). [ICH E2A]

Suspected Unexpected Serious Adverse Reactions (SUSARs)

The Sponsor-Investigator evaluates any SAE that has been reported regarding seriousness, causality and expectedness. If the event is related to the investigational product and is both serious and unexpected, it is classified as a SUSAR.

Assessment of Severity

The study site will grade the severity of adverse events experienced by study subjects according to the National Cancer Institute (NCI), Common Terminology Criteria for Adverse Events). Version 4.0 (published May 28, 2009). This document (referred to herein as the "NCI-CTCAE manual") provides a common language to describe levels of severity, to analyze and interpret data, and to articulate the clinical significance of all AEs. Adverse event general grade definitions are summarized as follows:

**Grade 1**	**Mild**	Transient or mild discomforts (< 48 hours), no or minimal medical intervention/therapy required, hospitalization not necessary (non-prescription or single-use prescription therapy may be employed to relieve symptoms, e.g., aspirin for simple headache, acetaminophen for post-surgical pain).
**Grade 2**	**Moderate**	Mild to moderate limitation in activity some assistance may be needed; no or minimal intervention/therapy required, hospitalization possible.
**Grade 3**	**Severe**	Marked limitation in activity, some assistance usually required; medical intervention/therapy required hospitalization possible.
**Grade 4**	**Life-threatening**	Extreme limitation in activity, significant assistance required; significant medical/therapy intervention required hospitalization or hospice care probable.
**Grade 5**	**Death**	Death.

Adverse events, not included in the NCI-CTCAE, will be recorded and graded 1 to 5 according to the General Grade Definition provided above.

10.1.2 Reporting of serious adverse events (SAE) and other safety related events

Reporting of SAEs

All SAEs must be reported immediately and within a maximum of 24 hours to the Sponsor-Investigator of the study and the Data Safety Monitoring Committee. The Sponsor-Investigator and the Data Safety Monitoring Committee will re-evaluate the SAE. SAEs resulting in death are reported to the local Ethics Committee (via local Investigator) within 7 days.

Reporting of SUSARs

A SUSAR needs to be reported to the local Ethics Committee (local event via local Investigator) and to Swissmedic for category B and C studies (via Sponsor-Investigator) within 7 days, if the event is fatal, or within 15 days (all other events).

Reporting of Safety Signals

All suspected new risks and relevant new aspects of known adverse reactions that require safety-related measures, i.e. so called safety signals, must be reported to the Sponsor-Investigator within 24 hours. The Sponsor-Investigator must report the safety signals within 7 days to the local Ethics Committee (local event via local Investigator) and to Swissmedic in case of a category B or C study.

Reporting and Handling of Pregnancies

Pregnant participants must immediately be withdrawn from the clinical study. Any pregnancy during the treatment phase of the study and within 30 days after discontinuation of study medication will be reported to the Sponsor-Investigator within 24 hours. The course and outcome of the pregnancy should be followed up carefully, and any abnormal outcome regarding the mother or the child should be documented and reported.

Periodic reporting of safety

An annual safety report is submitted once a year to the Data Safety Monitoring Committee. If necessary, this will be forwarded to the Local Ethics Committee and Swissmedic.

10.1.3 Follow up of (Serious) Adverse Events

As the study intervention only occurs within the first year post-transplant, AE and SAE are only collected within this time frame. If participants prematurely leave the study, AE and SAE will be collected until the one-year follow-up. Loss to follow-up within the first year-post-transplant is extremely rare and very unlikely to occur.

11. STATISTICAL METHODS

11.1 Hypothesis

In this proposal we will test the hypothesis that early treatment of rejection detected by urine CXCL10 monitoring will improve graft outcomes compared to standard monitoring by serum creatinine.

11.2 Determination of Sample Size

The sample size calculation is based on the primary 1-year composite endpoint. The primary 1-year composite outcome consists of at least one of the following four outcomes:

- Graft loss not due to death of the patient (anticipated incidence 2%)
- Biopsy-proven clinical acute rejection from 4-weeks to 1-year post-transplant (anticipated incidence 13-14%)
- Subclinical T-cell mediated rejection in 1-year surveillance biopsy (anticipated incidence 15%)
- Interstitial fibrosis / tubular atrophy with inflammation (IFTA+i) in 1-year surveillance biopsy (anticipated incidence 9.5%)

Thus, the estimated 1-year incidence of the primary composite outcome is ~ 40%.

We consider a 50% reduction of the primary outcome as clinically significant. The large effect size was assumed necessary to be clinically acceptable, given that urine CXCL10 monitoring is likely to increase the overall rates of biopsies and rejection treatments – interventions with potential risks to patients.

The sample size estimates were based on two-sample test of proportions using JMP 12 statistical software (www.jmp.com). Therefore, with an alpha error 0.05, power 80%, we need to enroll 81 patients in each arm. Based on experience from our centers, we conservatively assume a 10% drop-out rate. Therefore, the final sample size consists of 178 patients. Notably, this calculation is valid for the normal risk transplants.

During the recruitment phase of approximately 4 years, we will also include about 60 high risk transplants, which will be randomized and analyzed separately. The effect size of the intervention in this population is unknown but likely lower. Therefore, we regard this part of the study for this distinct group as a pilot trial.

11.3 Statistical criteria of termination of trial

Satisfaction of any of the following criteria at any time during the post-transplant (treatment) follow-up will trigger an ad-hoc Data Safety Monitoring Committee review:

- Any single occurrence of a life-threatening or fatal AE that is probably or definitely related to a study mandated procedure.

Across both Treatment Arms, incidence of:

- Biopsy-related bleeds or complications in **10%** or more
- PTLD in **1%** or more subjects
- Death in **5%** or more subjects

Within either treatment arms, incidence of:

- Normal histology on chemokine-guided biopsy in **50%** or more subjects
- Infection of any type requiring hospitalization in **40%** or more subjects
- Graft loss in **10%** or more subjects
- Indication biopsy-proven clinical rejection in **25%** or more subjects

11.4 Planned Analyses

11.4.1 Datasets to be analysed, analysis populations

As previously described, there will be two patient populations (normal risk transplants and high risk transplants). These dataset will be analysed separately but in the same manner.

11.4.2 Primary Analysis

The primary analysis will be performed on the intention-to-treat (ITT) population in the intervention vs the control arm. The ITT population isa defined as all randomized participants regardless of whether they complied with the chemokine screening or not. Participants will be assigned to the intervention groups and analyzed as randomized. The ITT population shall be used for analysis of all study endpoints except safety.

The initial analysis will be based on a binomial test for two proportions for the primary composite outcome in the two treatment arms. Patient survival from 1-12 months post-transplant is very high (>98%), so we anticipate that death with function will not impact the analysis. If missing outcome data is greater than anticipated, we will impute them according to FDA recommendations (44). The analysis will be done by the PI's with advice/help of the biostatistician after completion of the study.

11.4.3 Secondary Analyses

The secondary analyses will be done by the PI's with advice/help of the biostatistician after completion of the study. The applied statistical tests depend on the investigated outcome/parameter. We list some of them.

Creatinine Clearance/Glomerular Filtration Rate (eGFR): A linear mixed effects regression model will be used to assess the temporal evolution of eGFR with adjustment for covariates (e.g. age, gender, donor source, etc.) and a random subject effect to account for the within-subject correlations (repeated measures). Graft function will be evaluated as:

1. Change in graft function (eGFR) from 6-12, 6-24 and 6-60 months (slope, Δ).
2. Graft function (eGFR) at 6, 12, 24 and 60 months (absolute, mL/min).

Development of microvascular inflammation from implantation to 1-year (Banff ptc, g, c4d, cg): Change in injury levels (Banff scores) will be assess by paired-data analysis.

Development of IFTA from implantation to 1-year (Banff ci, ct, cv): Change in injury levels will be will be assessed by paired-data analysis.

Time from transplantation to clinical biopsy-proven acute rejection (ACR, AMR): will be summarized by the Kaplan Meier method and modeled using Cox regression analysis.

Proteinuria >500mg/day at 6, 12, 24 and 60 months post-transplant: Temporal trends in the progression of urine ACR will be modeled using linear mixed model analysis, with a random subject effect to account for the within-subject correlations due to within patient repeated measures.

Determine the kinetics of urinary chemokines in response to immunotherapy: Kinetics of urine CXCL10 will be characterized by descriptive statistics. We will use two different definitions for a significant response of urine CXCL10 levels to treatment: (i) a normalization of urine CXCL10/creatinine ≤1.00ng/mmol, or (ii) a reduction of CXCL10 >50% compared to pre-treatment levels. The urine CXCL10 response will then be correlated with clinical response to treatment and histological resolution of rejection in follow-up biopsies.

11.4.4 Interim analyses

No interim analysis for efficacy is planned.

11.4.5 Safety analysis

The collected safety parameters are collected as detailed in 9.2.2. as well as 10. The events will be counted and compared between the two study arms.

11.4.6 Deviation(s) from the original statistical plan

Deviation from the original statistical plan is very unlikely. We are unable to imagine and mention a justification.

11.5 Handling of missing data and drop-outs

Patient survival from 1-12 months post-transplant is very high (>98%), so we anticipate that death with function will not impact the analysis. If missing outcome data is greater than anticipated, we will impute them according to FDA recommendations (44).

12. QUALITY ASSURANCE AND CONTROL

12.1 Data handling and record keeping / archiving

12.1.1 Case Report Forms

An electronic Case Report Form is generated and maintained for each participant. Code identification will be used on the CRF. Only the PI's and authorized persons by the PI have access to the CRF and can enter data.

12.1.2 Specification of source documents

Source documents such as Informed Consent Forms and randomisation numbers are collected in paper form in dedicated binders at the office of the PI. Other source documents such a demographic data, histology reports, laboratory values etc. are available in electronic form in the Patient and Laboratory Information System at the University Hospital Basel. In addition, most of these data are available in the Patient Chart (=Flowsheet).

12.1.3 Record keeping / archiving

All study data and bio-banked samples must be archived for a minimum of 10 years after study termination or premature termination of the clinical trial. The study data are stored in a dedicated database located on servers at the University Hospital Basel, the bio-banked samples are stored in freezers at the University Hospital Basel and/or Department of Biomedicine.

12.2 Data management

12.2.1 Data Management System

We will use a custom-built Access-Database developed by the PI. The database is located on servers of the University Hospital Basel.

12.2.2 Data security, access and back-up

All PI's have access to the data. The database is password-protected. Once the data collection for the assessment of the primary outcome is completed, the database is closed. Regular backups of the database are performed.

12.2.3 Analysis and archiving

After completion of data collection, the primary dataset will be generated by a query in the database. The extracted data are stored on servers of the University Hospital Basel and further analysed with statistical software.

12.2.4 Electronic and central data validation

Missing data will trigger a review of the source data. Data validation will be performed when data are entered into the database (e.g. is the value within the expected data range etc.).

12.3 Monitoring

No specific monitoring by a person outside of the Data Safety Monitoring Committee is envisioned. The Data Safety Monitoring Committee will receive after 50, 100, and 150 patients, who completed the study the following data for review:

- All safety outcome (i.e. biopsy-related complications, immunosuppression-related complications)
- Number of allograft losses
- Number of death
- Frequency of normal histology on CXCL10-triggered biopsy
- Frequency of indication biopsy-proven clinical rejection

12.4 Audits and Inspections

No specific audits and inspections outside of the Data Safety Monitoring Committee is envisioned.

12.5 Confidentiality, Data Protection

The Data Safety Monitoring Committee has access to all study data for the purpose of monitoring and inspections. All PI's will have access to the protocol dataset, statistical code, etc during and after the study.

12.6 Storage of biological material and related health data

Samples, which are obtained in the context of this study (urine at specific time points; urine and serum at the time of allograft biopsies; allograft biopsy specimens) are stored for future investigations using an independent consent form "**Einwilligungserklärung für Weiterverwendung von biologischem Material und Daten**".

13. PUBLICATION AND DISSEMINATION POLICY

Data arising from this study is the property of the Sponsor, but it is the function of the study PI to disseminate information and make it available to the public. It is expected that the findings from this study will be presented at national and international scientific conferences and published in peer-reviewed journals.

14. FUNDING AND SUPPORT

14.1 Funding

This study is funded by SNF grant 32003B_169310 / 1

14.2 Other Support

Not applicable.

15. INSURANCE

An insurance certificate is required and will be attached as a document.

16. REFERENCES

1. El-Zoghby ZM, Stegall MD, Lager DJ, Kremers WK, Amer H, Gloor JM, and Cosio FG. Identifying specific causes of kidney allograft loss. Am J Transplant 9: 527-535, 2009.
2. Sellares J, de Freitas DG, Mengel M, Reeve J, Einecke G, Sis B, Hidalgo LG, Famulski K, Matas A, and Halloran PF. Understanding the causes of kidney transplant failure: the dominant role of antibody-mediated rejection and nonadherence. Am J Transplant 12: 388-399, 2012.
3. Wehmeier C, Georgalis A, Hirt-Minkowski P, Amico P, Hönger G, Vögele T, Brun N, Bock A, Wolff T, Gürke L, Bachmann A, Hopfer H, Dickenmann M, Steiger J, and **Schaub S**. 2222 kidney transplantations at the University Hospital Basel: a story of success and new challenges. (submitted) 2016.
4. Ekberg H, Tedesco-Silva H, Demirbas A, Vitko S, Nashan B, Gurkan A, Margreiter R, Hugo C, Grinyo JM, Frei U, Vanrenterghem Y, Daloze P, and Halloran PF. Reduced exposure to calcineurin inhibitors in renal transplantation. N Engl J Med 357: 2562-2575, 2007.
5. Moreso F, Ibernon M, Goma M, Carrera M, Fulladosa X, Hueso M, Gil-Vernet S, Cruzado JM, Torras J, Grinyo JM, and Seron D. Subclinical rejection associated with chronic allograft nephropathy in protocol biopsies as a risk factor for late graft loss. Am J Transplant 6: 747-752, 2006.
6. Rush D, Arlen D, Boucher A, Busque S, Cockfield SM, Girardin C, Knoll G, Lachance JG, Landsberg D, Shapiro J, Shoker A, and Yilmaz S. Lack of benefit of early protocol biopsies in renal transplant patients receiving TAC and MMF: a randomized study. Am J Transplant 7: 2538-2545, 2007.
7. Heilman RL, Devarapalli Y, Chakkera HA, Mekeel KL, Moss AA, Mulligan DC, Mazur MJ, Hamawi K, Williams JW, and Reddy KS. Impact of subclinical inflammation on the development of interstitial fibrosis and tubular atrophy in kidney transplant recipients. Am J Transplant 10: 563-570, 2010.
8. Amico P, Hirt-Minkowski P, Honger G, Gurke L, Mihatsch MJ, Steiger J, Hopfer H, and **Schaub S**. Risk stratification by the virtual crossmatch: a prospective study in 233 renal transplantations. Transpl Int 24: 560-569, 2011.
9. Nickerson P, Jeffery J, Gough J, McKenna R, Grimm P, Cheang M, and Rush D. Identification of clinical and histopathologic risk factors for diminished renal function 2 years posttransplant. J Am Soc Nephrol 9: 482-487, 1998.
10. Cosio FG, Grande JP, Wadei H, Larson TS, Griffin MD, and Stegall MD. Predicting subsequent decline in kidney allograft function from early surveillance biopsies. Am J Transplant 5: 2464-2472, 2005.
11. Moreso F, Carrera M, Goma M, Hueso M, Sellares J, Martorell J, Grinyo JM, and Seron D. Early subclinical rejection as a risk factor for late chronic humoral rejection. Transplantation 93: 41-46, 2012.
12. Loupy A, Vernerey D, Tinel C, Aubert O, Duong van Huyen JP, Rabant M, Verine J, Nochy D, Empana JP, Martinez F, Glotz D, Jouven X, Legendre C, and Lefaucheur C. Subclinical Rejection Phenotypes at 1 Year Post-Transplant and Outcome of Kidney Allografts. J Am Soc Nephrol 26: 1721-1731, 2015.
13. Liefeldt L, Brakemeier S, Glander P, Waiser J, Lachmann N, Schonemann C, Zukunft B, Illigens P, Schmidt D, Wu K, Rudolph B, Neumayer HH, and Budde K. Donor-specific HLA antibodies in a cohort comparing everolimus with cyclosporine after kidney transplantation. Am J Transplant 12: 1192-1198, 2012.
14. Hourmant M, Cesbron-Gautier A, Terasaki PI, Mizutani K, Moreau A, Meurette A, Dantal J, Giral M, Blancho G, Cantarovich D, Karam G, Follea G, Soulillou JP, and Bignon JD. Frequency and clinical implications of development of donor-specific and non-donor-specific HLA antibodies

after kidney transplantation. J Am Soc Nephrol 16: 2804-2812, 2005.

15. Wiebe C, Gibson IW, Blydt-Hansen TD, Karpinski M, Ho J, Storsley LJ, Goldberg A, Birk PE, Rush DN, and Nickerson PW. Evolution and clinical pathologic correlations of de novo donor-specific HLA antibody post kidney transplant. Am J Transplant 12: 1157-1167, 2012.
16. El TM, Grande JP, Keddis MT, Rodrigo E, Chopra B, Dean PG, Stegall MD, and Cosio FG. Kidney allograft survival after acute rejection, the value of follow-up biopsies. Am J Transplant 13: 2334-2341, 2013.
17. Rush D, Nickerson P, Gough J, McKenna R, Grimm P, Cheang M, Trpkov K, Solez K, and Jeffery J. Beneficial effects of treatment of early subclinical rejection: a randomized study. J Am Soc Nephrol 9: 2129-2134, 1998.
18. Kurtkoti J, Sakhuja V, Sud K, Minz M, Nada R, Kohli HS, Gupta KL, Joshi K, and Jha V. The utility of 1- and 3-month protocol biopsies on renal allograft function: a randomized controlled study. Am J Transplant 8: 317-323, 2008.
19. Hirt-Minkowski P, Amico P, Ho J, Gao A, Bestland J, Hopfer H, Steiger J, Dickenmann M, Burkhalter F, Rush D, Nickerson P, and **Schaub S**. Detection of clinical and subclinical tubulo-interstitial inflammation by the urinary CXCL10 chemokine in a real-life setting. Am J Transplant 12: 1811-1823, 2012.
20. **Schaub S**, Wilkins JA, Rush D, and Nickerson P. Developing a tool for noninvasive monitoring of renal allografts. Expert Rev Proteomics 3: 497-509, 2006.
21. Ho J, Wiebe C, Gibson IW, Rush DN, and Nickerson PW. Immune monitoring of kidney allografts. Am J Kidney Dis 60: 629-640, 2012.
22. Hirt-Minkowski P, Ho J, Gao A, Amico P, Koller MT, Hopfer H, Rush D, Nickerson P, and **Schaub S**. Prediction of long-term renal allograft outcome by early urinary CXCL10 chemokine levels. Transplantation Direct Epub 1-11-2015.
23. **Schaub S**, Nickerson P, Rush D, Mayr M, Hess C, Golian M, Stefura W, and Hayglass K. Urinary CXCL9 and CXCL10 levels correlate with the extent of subclinical tubulitis. Am J Transplant 9: 1347-1353, 2009.
24. Ho J, Rush DN, Karpinski M, Storsley L, Gibson IW, Bestland J, Gao A, Stefura W, HayGlass KT, and Nickerson PW. Validation of urinary CXCL10 as a marker of borderline, subclinical, and clinical tubulitis. Transplantation 92: 878-882, 2011.
25. Blydt-Hansen TD, Gibson IW, Gao A, Dufault B, and Ho J. Elevated urinary CXCL10-to-creatinine ratio is associated with subclinical and clinical rejection in pediatric renal transplantation. Transplantation 99: 797-804, 2015.
26. Rabant M, Amrouche L, Morin L, Bonifay R, Lebreton X, Aouni L, Benon A, Sauvaget V, Le VL, Aulagnon F, Sberro R, Snanoudj R, Mejean A, Legendre C, Terzi F, and Anglicheau D. Early low urinary CXCL9 and CXCL10 might predict immunological quiescence in clinically and histologically stable kidney recipients. Am J Transplant 2015.
27. Rabant M, Amrouche L, Lebreton X, Aulagnon F, Benon A, Sauvaget V, Bonifay R, Morin L, Scemla A, Delville M, Martinez F, Timsit MO, Duong van Huyen JP, Legendre C, Terzi F, and Anglicheau D. Urinary C-X-C Motif Chemokine 10 Independently Improves the Noninvasive Diagnosis of Antibody-Mediated Kidney Allograft Rejection. J Am Soc Nephrol 26: 2840-2851, 2015.
28. Hu H, Aizenstein BD, Puchalski A, Burmania JA, Hamawy MM, and Knechtle SJ. Elevation of CXCR3-binding chemokines in urine indicates acute renal-allograft dysfunction. Am J Transplant 4: 432-437, 2004.
29. Jackson JA, Kim EJ, Begley B, Cheeseman J, Harden T, Perez SD, Thomas S, Warshaw B, and Kirk AD. Urinary Chemokines CXCL9 and CXCL10 Are Noninvasive Markers of Renal Allograft Rejection and BK Viral Infection. Am J Transplant 11: 2228-2234, 2011.

30. Matz M, Beyer J, Wunsch D, Mashreghi MF, Seiler M, Pratschke J, Babel N, Volk HD, Reinke P, and Kotsch K. Early post-transplant urinary IP-10 expression after kidney transplantation is predictive of short- and long-term graft function. Kidney Int 69: 1683-1690, 2006.
31. Fairchild RL and Suthanthiran M. Urine CXCL10/IP-10 Fingers Ongoing Antibody-Mediated Kidney Graft Rejection. J Am Soc Nephrol 26: 2607-2609, 2015.
32. Ho J, Wiebe C, Gibson IW, Hombach-Klonisch S, Gao A, Rigatto C, Karpinski M, Storsley L, Nickerson PW, and Rush DN. Elevated urinary CCL2: Cr at 6 months is associated with renal allograft interstitial fibrosis and inflammation at 24 months. Transplantation 98: 39-46, 2014.
33. Ho J, Wiebe C, Rush DN, Rigatto C, Storsley L, Karpinski M, Gao A, Gibson IW, and Nickerson PW. Increased urinary CCL2: Cr ratio at 6 months is associated with late renal allograft loss. Transplantation 95: 595-602, 2013.
34. Ho J, Rush DN, Gibson IW, Karpinski M, Storsley L, Bestland J, Stefura W, HayGlass KT, and Nickerson PW. Early urinary CCL2 is associated with the later development of interstitial fibrosis and tubular atrophy in renal allografts. Transplantation 90: 394-400, 2010.
35. Hirt-Minkowski P, Rush D, Gao A, Hopfer H, Wiebe C, Nickerson P, **Schaub S**, and Ho J. Six-month urinary CCL2 and CXCL10 levels predict long-term renal allograft outcome. Transplantation (in press): 2016.
36. Stegall MD, Morris RE, Alloway RR, and Mannon RB. Developing New Immunosuppression for the Next Generation of Transplant Recipients: The Path Forward. Am J Transplant 2016.
37. Schold JD and Kaplan B. The elephant in the room: failings of current clinical endpoints in kidney transplantation. Am J Transplant 10: 1163-1166, 2010.
38. Haas M. The Revised (2013) Banff Classification for Antibody-Mediated Rejection of Renal Allografts: Update, Difficulties, and Future Considerations. Am J Transplant 2015.
39. Wehmeier C, Amico P, Hirt-Minkowski P, Georgalis A, Burkhalter F, Steiger J, Dickenmann M, Hopfer H, and **Schaub S**. Rejection phenotypes in the current era of immunosuppression and risk stratification. (in preparation) 2016.
40. Cosio FG, El TM, Cornell LD, Schinstock CA, and Stegall MD. Changing Kidney Allograft Histology Early Posttransplant: Prognostic Implications of 1-Year Protocol Biopsies. Am J Transplant 16: 194-203, 2016.
41. Shishido S, Asanuma H, Nakai H, Mori Y, Satoh H, Kamimaki I, Hataya H, Ikeda M, Honda M, and Hasegawa A. The impact of repeated subclinical acute rejection on the progression of chronic allograft nephropathy. J Am Soc Nephrol 14: 1046-1052, 2003.
42. Park WD, Griffin MD, Cornell LD, Cosio FG, and Stegall MD. Fibrosis with inflammation at one year predicts transplant functional decline. J Am Soc Nephrol 21: 1987-1997, 2010.
43. Mannon RB, Matas AJ, Grande J, Leduc R, Connett J, Kasiske B, Cecka JM, Gaston RS, Cosio F, Gourishankar S, Halloran PF, Hunsicker L, and Rush D. Inflammation in areas of tubular atrophy in kidney allograft biopsies: a potent predictor of allograft failure. Am J Transplant 10: 2066-2073, 2010.
44. Groenwold RH, Moons KG, and Vandenbroucke JP. Randomized trials with missing outcome data: how to analyze and what to report. CMAJ 186: 1153-1157, 2014.

17. APPENDICES

None

Pocket Guide for physicians

Chemokin-Studie

bei Fragen → Stefan 86736, Patricia 65622, Joelle 85736 (Labor)

Einschlusskriterien

- Erwachsene(r) Nierentransplantations-empfänger(in) mit unterschriebener Einverständniserklärung

Ausschlusskriterien

- HLA-identische Lebendnierenspende
- 'Primary non-function'
- Teilnahme an interventionellen Studien mit Immunsuppressiva

Randomisierung / Stratifizierung

- Normalrisiko
  - ➡ Interventionsarm
  - ➡ Kontrollarm
- Hochrisiko (=ABOi, HLA-DSA, Ehemann zu Ehefrau mit gemeinsamen Kindern)
  - ➡ Interventionsarm
  - ➡ Kontrollarm

Ohne Studienteilnahme wird das Protokollbiopsieschema (3-Mt, 6-Mt, ev. 12-Mt) befolgt.

Abstossungsbehandlung

Klinische Abstossungen (Empfehlung)

Borderline TCMR	Steroide p.o. oder i.v. 3-5x mit Tapering. Basis-IS beibehalten oder leicht erhöhen.
TCMR 1A, 1B	Steroide i.v. 3-5x mit Tapering. Basis-IS beibehalten oder leicht erhöhen.
TCMR 2A, 2B	Steroide i.v. 3-5x mit Tapering oder ATG. Basis-IS beibehalten oder erhöhen.
ABMR	Steroide i.v. +/- ATG +/- PP +/- Ivlg gemäss Schweregrad und Kontext. Basis-IS beibehalten oder erhöhen.

Subklinische Abstossungen (Empfehlung)

Borderline TCMR	Steroide p.o. mit Tapering. Basis-IS beibehalten oder leicht erhöhen.
TCMR 1A, 1B	Steroide p.o. oder i.v. 3-5x mit Tapering. Basis-IS beibehalten oder leicht erhöhen.
TCMR 2A, 2B	Steroide p.o. oder i.v. 3-5x mit Tapering oder ATG gemäss Schweregrad und Kontext. Basis-IS beibehalten oder erhöhen.
ABMR	Steroide p.o. oder i.v. 3-5x mit Tapering oder ATG gemäss Schweregrad und Kontext. Basis-IS beibehalten oder erhöhen.

Chemokin-Studie

bei Fragen → Stefan 86736, Patricia 65622, Joelle 85736 (Labor)

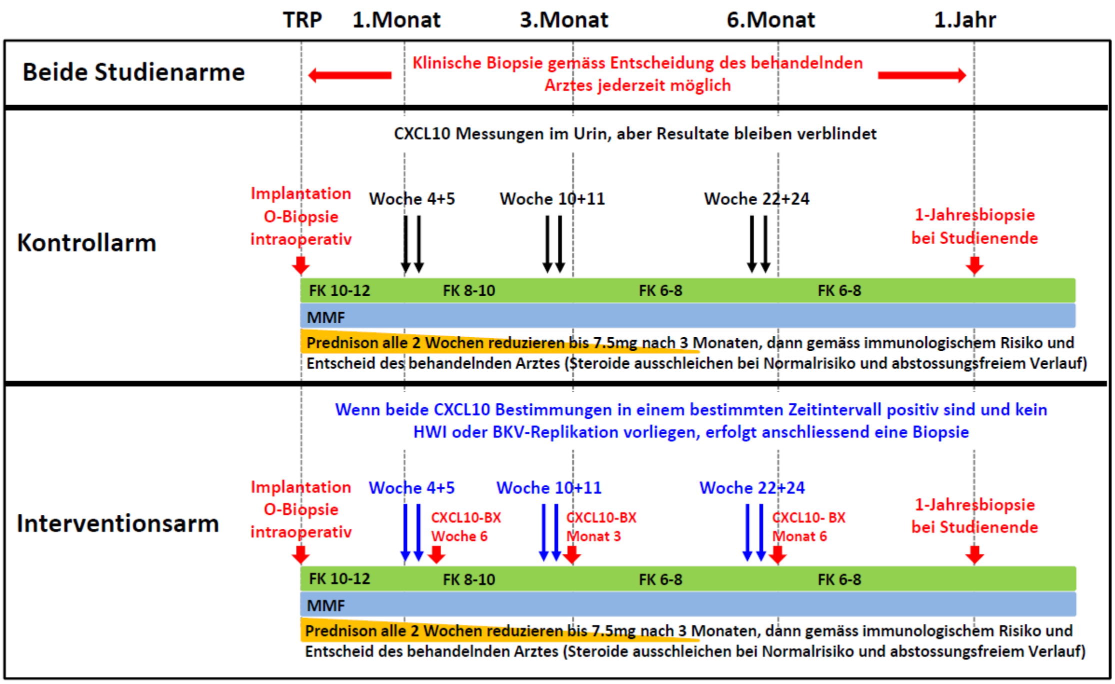

EKNZ

Ethikkommission
Nordwest- und
Zentralschweiz

Präsident
Prof. André P. Perruchoud
Vizepräsidenten
Prof. Gregor Schubiger
Dr. Marco Schärer

Prof. Stefan Schaub
University Hospital Basel
Transplantation Immunology &
Nephrology
Petersgraben 4
4031 Basel

Basel, 19. Juni 2017

Verfügung der Ethikkommission Nordwest- und Zentralschweiz (EKNZ)

**Project-ID**	2017-00742
**Projekttitel**	Urine CXCL10 chemokine monitoring post-renal transplant: a single-center randomized controlled trial
**Haupt-Prüfer / Koordinierender Prüfer**	Prof. Stefan Schaub
**Sponsor**	Haupt-Prüfer / Koordinierender Prüfer
**Zentren**	Prof. Stefan Schaub, Transplantation Immunology & Nephrology, Basel

I. Entscheidverfahren

☐ ordentliches Verfahren ☐ vereinfachtes Verfahren ☒ Präsidialverfahren

II. Entscheid

Prof. Stefan Schaub, Transplantation Immunology & Nephrology, Basel

- ☒ Die Bewilligung wird erteilt → die Auflagen der EKNZ vom 24. Mai 2017 wurden somit erfüllt.
- ☐ Die Bewilligung wird mit Auflagen erteilt
- ☐ Die Bewilligung kann noch nicht erteilt werden
- ☐ Die Bewilligung wird nicht erteilt
- ☐ Auf das Gesuch wird nicht eingetreten

III. Klassifizierung

☒ klinische Studie gemäss KlinV

Kategorie: B

- ☐ mit Arzneimitteln
- ☐ mit Transplantatprodukten
- ☐ mit genetisch veränderten oder pathogenen Organismen
- ☒ anderer klinischer Versuch gemäss Kapitel 4 KlinV
- ☐ Umkategorisierung gemäss Art. 71, Abs. 3, KlinV
- ☐ mit Medizinprodukten
- ☐ der Gentherapie
- ☐ der Transplantation
- ☐ mit ionisierender Strahlung

IV. Zusammensetzung der Kommission

	Name, Vorname	Berufliche Stellung / Titel	m	f	am Beschluss beteiligt ja	nein
**Vorsitz**	Prof. A. P. Perruchoud	Präsident der EKNZ	x	☐	x	☐

V. Gebühren

Tarifcode: **Betrag:** **CHF .--**
Gemäss der geltenden Gebührenordnung von swissethics.

VI. Rekursmöglichkeiten

Gegen diesen Entscheid kann an den Regierungsrat des Kantons Basel-Stadt (Rathaus, Marktplatz 9, 4051 Basel) rekurriert werden. Der Rekurs ist innert 10 Tagen seit Eröffnung des Entscheides bei der Rekursinstanz anzumelden; innert 30 Tagen, vom gleichen Zeitpunkt an gerechnet, ist die Rekursbegründung einzureichen, welche die Anträge und deren Begründung mit Angabe der Beweismittel zu enthalten hat. Bei völliger oder teilweiser Abweisung des Rekurses können die Kosten der Rekurrentin respektive dem Rekurrenten ganz oder teilweise auferlegt werden.

VII. Kopie an

☐ Swissmedic
☐ BAG
☐ Andere

Unterschriften

i.V. [signature]
Prof. Dr. med. André P. Perruchoud
Präsident

Pflichten des Gesuchstellers (Sponsor oder Prüfer):

Einreichung Dokumente: revidierte Dokumente und neue Dokumente zur Studie/zum Projekt sollen ausschliesslich über das Web-Portal BASEC eingereicht werden, auf der entsprechenden Formularseite des betreffenden Gesuches. Obsolete Dokumente sind dabei zu entfernen und Datums- und Versionsangaben entsprechend zu ergänzen. Die erfolgten Änderungen müssen im Korrekturmodus abgefasst werden und zusätzlich als ‚clean'-Version eingereicht werden. Die Studieninformationen und -einwilligungen, das Protokoll und die Amendments müssen in durchsuchbaren PDF-Dateien eingereicht werden, insbesondere müssen gescannte Dokumente eine Texterkennung durchlaufen haben (OCR). Das unterschriebene und datierte Begleitschreiben muss die Antworten auf eventuell von der EK gestellte Fragen enthalten. Revidierte Dokumente sind auch den weiteren Zulassungsbehörden zuzustellen, sofern diese involviert sind.

Anmerkung: Die zuständige Ethikkommission überprüft im Rahmen des Bewilligungsverfahrens Aufklärungsbogen und Einwilligungserklärung in einer der Amtssprachen Deutsch, Französisch oder Italienisch. Aufklärungsbogen und Einwilligungserklärung in einer anderen Sprache werden von der Ethikkommission lediglich zur Kenntnis genommen. Für die korrekte Übersetzung ist der Sponsor oder die Projektleitung verantwortlich.

Meldepflichten: Die rechtlich bindenden Melde- resp. Bewilligungspflichten an die Ethikkommission für wesentliche Änderungen, einen vorzeitigen Studienabbruch, unerwünschte Ereignisse u.a. sind einzuhalten (Verordnungen des Bundes). Der Abschlussbericht ist spätesten ein Jahr nach Studienende der Ethikkommission einzureichen.

Registrierungspflicht: Der Sponsor muss - falls es sich um einen klinischen Versuch handelt – diesen in einem WHO-Primärregister oder im Register der Nationalen Medizinbibliothek der USA (clinicaltrials.gov) erfassen und anschliessend diese Nummer im BASEC-Portal eingeben. Die Übertragung der erforderlichen Daten in das Swiss National Clinical Trials Portal (SNCTP) kann nach Bewilligung der Ethikkommission und Zustimmung des Gesuchstellers automatisch erfolgen. Die Informationen über den klinischen Versuch sind in beiden Registern öffentlich zugänglich. Zusätzlich veröffentlicht swissethics wenige Informationen wie Titel, Projekttyp oder Leit-Ethikkommission aller durch die kantonalen Ethikkommissionen bewilligten Gesuche auf swissethics.ch (ausser Phase-I-Studien).

Die Ethikkommission bestätigt, dass sie nach ICH-GCP arbeitet.

Anmerkung: detaillierte Anleitungen zur Einreichung auf BASEC befinden sich im Portal selbst.

Anhang

- Liste eingereichter Dokumente, Stand 15. Juni 2017

Bedeutung der möglichen Entscheide

Die Bewilligung wird erteilt: Das Vorhaben gemäss bewilligtem Forschungsplan kann gestartet und im Rahmen der anwendbaren rechtlichen Bestimmungen durchgeführt werden.
Bewilligungen für klinische Versuche mit Heilmitteln der Kategorie B und C stehen unter dem Vorbehalt, dass

1. allfällig durch die zuständige eidgenössische Zulassungsbehörde (Swissmedic/BAG) festgestellte Mangel keine Änderungen der von der Ethikkommission evaluierten Unterlagen erfordern, und dass
2. die Bewilligung der eidgenössischen Zulassungsbehörde (Swissmedic/BAG) vorliegt.

Anhang

Eingereichte Dokumente für das Hauptzentrum

Prof. Stefan Schaub, Transplantation Immunology & Nephrology, Basel		
Dokument	Dok.Datum	Version
00_Antworten.pdf	14.06.2017	
01_Study_Protocoll_Synopsis_V2.0_2017-06-14 - clean.pdf	14.06.2017	2.0
01_Study_Protocoll_Synopsis_V2.0_2017-06-14.pdf	14.06.2017	2.0
03_Studieninformation_V2.0_2017-06-14 - clean.pdf	14.06.2017	2.0
03_Studieninformation_V2.0_2017-06-14.pdf	14.06.2017	2.0
02_Study_Protocol_V2.0_2017-06-14 - clean.pdf	14.06.2017	2.0
02_Study_Protocol_V2.0_2017-06-14.pdf	14.06.2017	2.0
04_CRF_Parameterliste_V2.0_2017-06-14 - clean.pdf	14.06.2017	2.0
04_CRF_Parameterliste_V2.0_2017-06-14.pdf	14.06.2017	2.0
GCP-Basiskurs-Zertifikat.pdf	14.06.2017	
Bestätigung zur Anmeldung des GCP-Aufbaukurses ....pdf	14.06.2017	
snf-verfugung-2016-09-21.pdf	21.09.2016	
17.016-versicherungszertifikat.pdf	05.05.2017	
05-biobanken-reglement-v1.0-2017-06-14.pdf	14.06.2017	

Final protocol and summary of changes

The only changes in the final protocol, compared to the original protocol, apply to the outcome definitions.

The original outcomes in 2017 were:

The primary 1-year composite outcome will consist of at least one of the following four outcomes:

- Graft loss not due to death of the patient
- Biopsy-proven clinical acute rejection from 4-weeks to 1-year post-transplant
- Subclinical T-cell mediated rejection in 1-year surveillance biopsy
- Interstitial fibrosis / tubular atrophy with inflammation (IFTA+I defined by the Mayo Clinic criteria) in 1-year surveillance biopsy

Secondary Outcomes

- Microvascular inflammation at 1-year (ptc, g, c4d, cg)
- Development of IFTA from implantation to 1-year (Δ ci, ct, cv)
- Days from transplantation to biopsy-proven clinical acute rejection
- Proteinuria >500mg/day at 6- and 12-months post-transplant
- total number of biopsies, indication for biopsy and CXCL10-triggered biopsies within the first year post-transplant
- biopsy-related complications within the first year post-transplant biopsy-related complications within the first year post-transplant
- immunosuppression-related complications as infections and cancer within the first year post-transplant

The final outcomes were (changes marked with yellow background):

The primary 1-year composite outcome will consist of at least one of the following four outcomes:

- Graft loss not due to or death of the patient
- Biopsy-proven clinical acute rejection from 4-weeks to 1-year post-transplant
- Subclinical ~~T-cell mediated~~ rejection in 1-year surveillance biopsy
- ~~Interstitial fibrosis / tubular atrophy with inflammation (IFTA+I defined by the Mayo Clinic criteria) in 1-year surveillance biopsy~~ --> replaced by chronic active TCMR according to Banff 2019 classification
- NEW: Development of de novo DSA
- NEW: Estimated eGFR <25ml/min at one-year posttransplant

Secondary Outcomes

- ~~Microvascular inflammation at 1-year (ptc, g, c4d, cg)~~ is now included in primary outcome as "Subclinical rejection in 1-year surveillance biopsy"
- Development of IFTA from implantation to 1-year (Δ ci, ct, cv)
- Days from transplantation to biopsy-proven clinical acute rejection
- Proteinuria >500mg/day at 6- and 12-months post-transplant
- total number of biopsies, indication for biopsy and CXCL10-triggered biopsies within the first year post-transplant
- biopsy-related complications within the first year post-transplant biopsy-related complications within the first year post-transplant

- immunosuppression-related complications as infections and cancer within the first year post-transplant

The chances in the outcomes were strongly considered as necessary by the PI and the Monitoring Committee to adapt to emerging knowledge. Notably, all outcomes in the original protocol are part of the final protocol. The individual justifications for the chances are as follows:

- Subclinical rejection in 1-year surveillance biopsy including ABMR phenotypes
  ABMR is not frequent within the first year posttransplant, but associated with a worse prognosis (1-3). Therefore, it is meaningful to include all rejection phenotypes in the primary outcome, not only TCMR. ABMR phenotypes detected in 1-year surveillance biopsies (secondary outcome in the primary protocol) were compiled into the primary outcome "Subclinical rejection in 1-year surveillance biopsy".
- IFTA+i --> chronic active TCMR
  Chronic active TCMR was introduce in the Banff 2017 classification (published 01/2018) (4). To comply with the current nomenclature, we adopted chronic active TCMR instead of IFTA+i.
- NEW: Development of de novo DSA
  The incidence of de novo DSA development within the first year is between 2-10%. De novo DSA are associated with a poor outcome (1, 3, 5).
- NEW: Estimated eGFR <25ml/min at one-year posttransplant
  A recent study in a large population demonstrated that an eGFR<25/ml is associated with a very poor 5-year graft survival (48% vs 85%; p<0.001) (6)

References

1. Loupy A, Aubert O, Orandi BJ, Naesens M, Bouatou Y, Raynaud M, et al. Prediction system for risk of allograft loss in patients receiving kidney transplants: international derivation and validation study. BMJ. 2019;366:l4923.
2. Schinstock CA, Mannon RB, Budde K, Chong AS, Haas M, Knechtle S, et al. Recommended Treatment for Antibody-mediated Rejection After Kidney Transplantation: The 2019 Expert Consensus From the Transplantion Society Working Group. Transplantation. 2020;104(5):911-22.
3. Wiebe C, Gibson IW, Blydt-Hansen TD, Pochinco D, Birk PE, Ho J, et al. Rates and determinants of progression to graft failure in kidney allograft recipients with de novo donor-specific antibody. Am J Transplant. 2015;15(11):2921-30.
4. Haas M, Loupy A, Lefaucheur C, Roufosse C, Glotz D, Seron D, et al. The Banff 2017 Kidney Meeting Report: Revised diagnostic criteria for chronic active T cell-mediated rejection, antibody-mediated rejection, and prospects for integrative endpoints for next-generation clinical trials. Am J Transplant. 2018;18(2):293-307.
5. Raynaud M, Aubert O, Reese PP, Bouatou Y, Naesens M, Kamar N, et al. Trajectories of glomerular filtration rate and progression to end stage kidney disease after kidney transplantation. Kidney Int. 2021;99(1):186-97.
6. Wehmeier C, Amico P, Sidler D, Wirthmuller U, Hadaya K, Ferrari-Lacraz S, et al. Pre-transplant donor-specific HLA antibodies and risk for poor first-year renal transplant outcomes: results from the Swiss Transplant Cohort Study. Transpl Int. 2021;34(12):2755-68.

Final statistical analysis plan

The original statistical analysis plan was described in the original protocol. The final statistical analyses plan includes more details.

General reporting of the study
The study will be reported according to the CONSORT statement. The CONSORT checklist and the CONSORT flow diagram will be used.

Descriptive analysis and comparison of variables
Categorical data will be reported as counts (percentage) and analyzed by chi-squre test. Continuous data will be reported as median (interquartile range) or mean ± standard deviation as appropriate, and compared by the Wilcoxon-test or the t-test, respectively.

Primary outcome analysis
In addition to the intention-to-treat analysis, a modified intention-to-treat and a per-protocol analysis will be performed. The modified intention-to-treat analysis includes only patients, who had an adequate one-year surveillance biopsy. The per-protocol analysis will include only patients, who fully complied with the protocol (i.e. all CXCL10 monitoring samples were available and analyzed, all CXCL10-triggered biopsies were performed, and all CXCL10-detected rejection episodes were treated according to the protocol) and had an adequate one-year surveillance biopsy. The primary outcome analysis will be based on a comparison of two proportions calculated as a relative risk with 95% confidence intervals. As some outcomes are expected to have low numbers, the two-tailed Fishers exact test p-value will be reported.

Secondary outcome analysis
The analyses will be performed as detailed in the description and comparison of variables section above. The incidence of rejection between the two arms will be compared by a time-to-event analysis (Kaplan-Meier method) and compared by the log-rank test.

Additional analyses
We have previously reported that the urine CXCL10 burden is a strong predictor for long-term graft outcome (Hirt-Minkowski P et al. Prediction of Long-term Renal Allograft Outcome By Early Urinary CXCL10 Chemokine Levels. Transplantation Direct, 2015; PMID: 27500231). A similar analysis will be performed in this study. The urine CXCL10 burden (mean of all measurements at the three monitoring time points) will be calculated and tertiles (or quartiles) correlated with allograft rejection in one-year surveillance biopsies using the Cochran-Armitage trend-test.

To assess the diagnostic accuracy of urine CXCL10 for prediction of allograft rejection, the AUC will be determined in a ROC analysis. This analysis will include all allograft biopsies with a concurrent urine CXCL10 measurement and no confounders (UTI or BKPyV infection).

Summary of changes in the statistical analysis plan

- The only major change in the final statistical analysis plan, compared to the original statistical analysis plan, concerns the immunological high risk group. This group will be merged with the standard risk patients into their respective randomization group (intervention or control arm). As they were randomized in strata, we anticipated no bias in subsequent analyses. To be consistent with the original statistical analysis plan, we will investigate the two immunological risk groups separately in a sensitivity analysis.
- The Banff classification for allograft histology is subject to changes following emerging evidence from published studies and discussions among experts. When the CXCL10 trial was planned and initiated (year 2017), the Banff 2015 version was the most recent one. We adhered to this version throughout the whole study for all histological allograft assessments. As the Banff 2019 version had significant changes (e.g. 'borderline' changes are required to have an i-score>0), we will report the outcomes using the Banff 2019 definitions as well. However, all treatment decisions during the study were based on the Banff 2015 definitions.
